# Geometry-Associated Mechanical Trends of Graphene Oxide and Carbon Nanotube SPEEK Sizings in CF/PEEK Commingled Yarn Laminates

**DOI:** 10.3390/polym18202565

**Published:** 2026-10-18

**Authors:** Xuejiang Yu, Yousi Chen, Ai Lu

**Affiliations:** 1 National Key Laboratory of Chemical Explosion Safety, Institute of Chemical Materials, China Academy of Engineering Physics (CAEP), Mianyang 621900, China; 2 School of Materials Science and Engineering, Dalian University of Technology, Dalian 116024, China

**Keywords:** carbon fiber, PEEK, SPEEK sizing, graphene oxide, carbon nanotube, interfacial modification, polymer composite

## Abstract

The interfacial region dictates the load-transfer efficiency and mechanical performance of continuous carbon fiber/polyetheretherketone (CF/PEEK) thermoplastic composites. Nanofiller geometry represents a promising route to tailor fiber–matrix interphases, yet it remains challenging to decouple geometric effects from confounding variables including surface chemistry, defect density, and filler dispersion. In this work, sulfonated poly(ether ether ketone) (SPEEK) sizing modified by one-dimensional (1D) carbon nanotubes (CNT) and two-dimensional (2D) graphene oxide (GO) was deposited onto carbon fibers to construct geometry-differentiated interphases. A Box–Behnken response-surface methodology was adopted to identify the optimal hot-pressing processing window. Low-porosity laminates with consistent fiber volume fractions were fabricated to minimize void-induced property scatter. Mechanical characterizations reveal that both GO and CNT modified sizings substantially improve interlaminar shear strength and flexural/tensile performance relative to desized reference samples. SEM fractography confirms a transition from pure adhesive interfacial debonding toward mixed cohesive/adhesive failure upon nanofiller incorporation. Halpin–Tsai semi-quantitative estimation demonstrates that the direct stiffening contribution from the intrinsic rigidity of nanofillers is negligible. We therefore propose a working hypothesis that the enhanced mechanical properties arise from geometry-associated interphase modification, where nanofiller morphology alters polymer chain mobility and local crystallization behavior at the fiber–matrix boundary. This screening study highlights the potential of nanofiller geometric design for interphase engineering in CF/PEEK composites, while also clarifying current limitations: geometric, chemical, and dispersion effects cannot be fully isolated under the present experimental framework, and dedicated interfacial characterization is required in future work to validate the proposed interphase mechanisms.

## 1. Introduction

Polyetheretherketone (PEEK) and other polyaryletherketone (PAEK) thermoplastic matrices are increasingly considered for aerospace and automotive structural parts because of their toughness, chemical resistance, and high service temperature [1,2]. Continuous carbon fiber (CF) reinforced PEEK laminates, however, remain confined to secondary or non-critical load-bearing applications due to two coupled limitations: (i) the high melt viscosity of PEEK (300–500 Pa·s at 380 °C [3]) and its chemical inertness make melt impregnation and interfacial bonding difficult, and (ii) the conventional epoxy-based sizings on as-received CF are thermally labile at the 350–400 °C processing window of PEEK and are intrinsically immiscible with the PEEK matrix [4]. Commingled yarn architectures, in which reinforcing fibers and thermoplastic fibers are blended at the yarn scale, shorten the resin flow path and improve prepreg flexibility [4,5,6]. The remaining bottleneck is the CF/PEEK interface.

SPEEK, which retains the PEEK main chain while introducing polar –SO_3_H groups, has emerged as a promising water-based sizing agent for CF: it offers good thermal stability, water solubility, and structural compatibility with the PEEK matrix [7,8]. Because SPEEK and the matrix share the same PEEK backbone, the chemical similarity between the SPEEK overlayer and the PEEK matrix is maximized, providing a robust platform for subsequent nanofiller deposition [9]. Polymer-only SPEEK sizings improve the interlaminar shear strength (ILSS) of CF/PEEK by 17–26% over the desized baseline [7,10,11], but the absolute gain is limited because a soft polymer shell cannot fully arrest interlaminar crack propagation under severe shear. A second reinforcement tier—rigid nanofillers—is required to construct a true multi-scale interphase.

Among rigid nanofillers, two structural families dominate the literature: 2D GO, with high specific surface area and lamellar morphology [12,13], and 1D CNT [14], with high aspect ratio and tube-like geometry [15,16]. Both improve interfacial load transfer through physical anchoring and chemical effects. Direct side-by-side comparison under an identical polymer matrix and identical processing conditions is, however, rare: most studies compare one of these families against an unmodified baseline, often at a single concentration, and the question of “which geometry for which property” remains open [17].

The present work addresses this gap by performing, in a woven-fabric CF/PEEK system, a side-by-side comparison of 2D GO and 1D CNT co-deposited with an identical water-based SPEEK matrix at three concentrations (0.5, 1.0, and 2.0 g·L^−1^). We examine whether the two geometries are associated with differentiated mean mechanical signatures: 2D face-to-face contact of GO with a higher mean in-plane stiffness and 1D line-to-face bridging of CNT with higher mean strength values. We present the resulting geometry-associated contrast as a working hypothesis consistent with the present single-plate dataset, not as a demonstrated mechanism or a validated selection rule, and we note that the present n = 5 within-plate sampling and the layered interphase evidence (FTIR, XPS, TGA) do not support a stronger claim [18].

## 2. Experimental

### 2.1. Materials

Continuous CF (HF 40A, 6K, T700 grade; Jiangsu Hengshen Co., Ltd., Danyang, China) had a linear density of 2230 dtex, density of 1.78 g·cm^−3^, single-filament diameter of ≈5 μm, tensile strength of 5600 MPa, and tensile modulus of 295 GPa; the surface was coated with a commercial epoxy sizing. PEEK fiber (Changzhou Chuangying New Material Technology Co., Ltd., Changzhou, China) had a linear density of 611 dtex, density of 1.30 g·cm^−3^, and single-filament diameter of ≈20 μm. PEEK powder (Jilin University Special Plastics Engineering Research Co., Ltd., Jilin, China) was used for SPEEK synthesis. Acetone (≥99.5%), concentrated H_2_SO_4_ (98%), multilayer GO (5–10 layers, 5–50 μm diameter; Suzhou Carbon Graphene Technology Co., Ltd., Suzhou, China), and multi-walled CNT (diameter < 8 nm, length 10–30 μm, purity > 98%; Chengdu Organic Chemistry Co., Ltd., Chinese Academy of Sciences, Chengdu, China) were used as received. Deionized water was laboratory-made.

### 2.2. Synthesis of SPEEK

PEEK powder (3 g) was vacuum-dried at 80 °C for 8 h and dissolved in 100 mL of pre-cooled 98% H_2_SO_4_. Sulfonation was carried out at 45 °C for 6 h under continuous stirring. The product was precipitated in ice-cold deionized water, washed repeatedly until the filtrate pH reached 7, and vacuum-dried at 50 °C for 12 h to yield a light-yellow SPEEK master batch. The sulfonation degree (DS) was determined by acid–base titration (phenolphthalein, 0.01 mol·L^−1^ NaOH in DMF) following the procedure of Xing et al. [19] and was 0.78, within the 0.6–1.2 range required for water solubility and storage stability. The target DS balances water solubility (DS ≥ 0.6) against excessive swelling and moisture uptake (DS > 1.2); the present value of 0.78 is selected for its stability trade-off rather than for its maximum polar-group density. Higher DS would risk increased water uptake during the long drying step (60 °C, 2 h) and plasticization of the SPEEK overlayer, which would weaken rather than strengthen the interphase.

### 2.3. Surface Treatment

The as-received commingled yarn fabric was first desized by refluxing in acetone at 70 °C for 24 h, then rinsed with deionized water and dried. For the SPEEK-only series, the desized fabric was immersed in 0.0025, 0.005, or 0.010 g·mL^−1^ SPEEK solutions for 2 h and slowly dried at 60 °C. The 0.010 g·mL^−1^ SPEEK concentration was selected as the practical upper-limit working concentration for fabric-scale impregnation. Although the DS ≈ 0.78 SPEEK polymer remains macroscopically water-soluble at 0.015 g·mL^−1^, this higher-concentration solution displays substantially increased viscosity. Trial impregnation tests revealed that elevated viscosity impairs capillary wicking and uniform penetration into inter-yarn voids of the woven commingled-yarn fabric, producing patchy coverage and local over-deposition on the textile surface. Hence, 0.010 g·mL^−1^ was chosen to guarantee homogeneous sizing distribution across the whole fabric [20,21,22]. Higher concentrations are limited by processing-related viscosity-driven inhomogeneity, not by absolute thermodynamic solubility of SPEEK in water. For the nanocomposite series, GO or CNT was dispersed into the 0.010 g·mL^−1^ SPEEK solution by ultrasonication (500 W, 40 kHz, 30 min) to give three concentrations: 0.5, 1.0, and 2.0 g·L^−1^. Both sides of the fabric were immersed in the sizing solution to ensure uniform coverage; after immersion, the fabric was dried at 60 °C in an air oven until constant weight (≈2 h). The 11 specimens are listed in Table 1.

### 2.4. Laminate Fabrication

**Figure 1:**
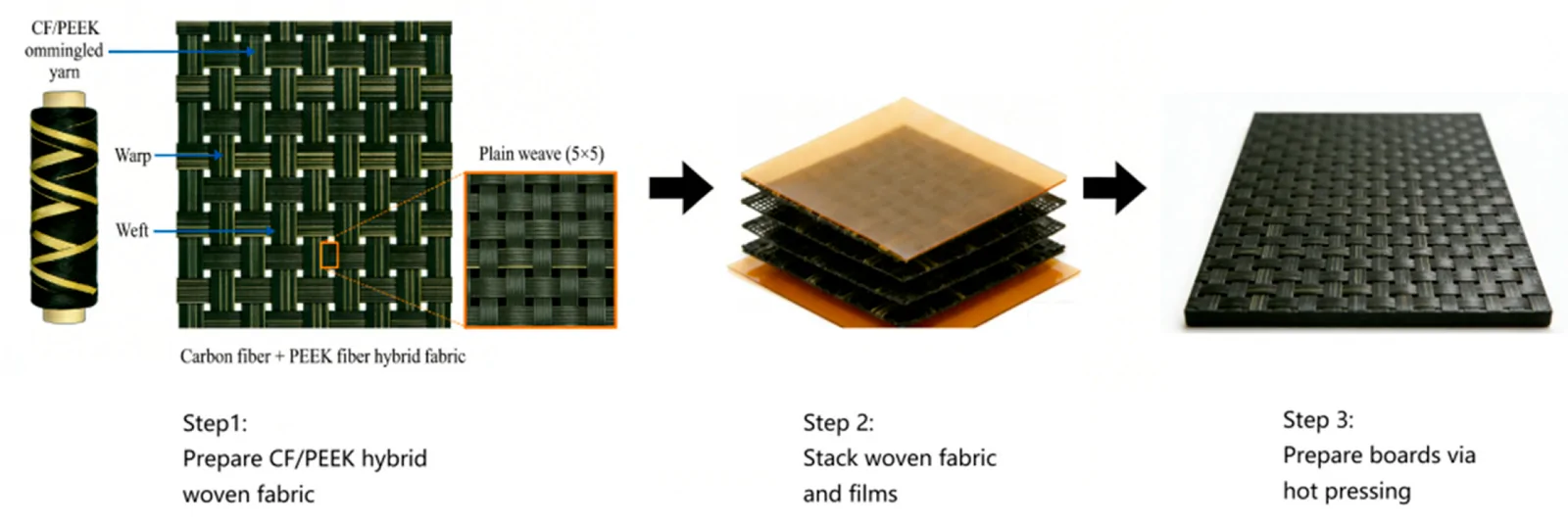
Architecture of the CF/PEEK commingled yarn plain-woven fabric laminate. The warp and weft densities are both 5 ends·cm^−1^; ten plies are stacked and hot-pressed at 350 °C/2.0 MPa/20 min to give a 2.0 ± 0.1 mm laminate with 64.60 wt% carbon fiber.

A custom-made 2D plain-woven commingled-yarn fabric (warp and weft densities both 5 ends·cm^−1^; areal density 345 g·m^−2^; theoretical thickness 0.219 mm) was supplied by Ningbo Panling Industrial Co., Ltd. (Ningbo, China) as the consolidated-fabric feedstock. **The architecture of this CF/PEEK commingled yarn plain-woven fabric laminate is illustrated in Figure 1.** The fabric is composed of side-by-side commingled yarns in which one CF bundle is adjacent to PEEK fiber strands (no twisting or wrapping), and the target carbon fiber mass fraction in the consolidated laminate is 64.60 wt%. The as-received fabric was cut into 300 × 300 mm^2^ plies, stacked in a [0/90]_10_ sequence (ten plies), sandwiched between polyimide (PI) films, and hot-pressed at 350 °C/2.0 MPa/20 min in a laboratory press (P300E). This hot-pressing window was selected from a Box–Behnken optimization (15 runs; S1 Tables S1–S3 and Figures S1 and S2) that gave R^2^ = 0.9905 for flexural strength and R^2^ = 0.9817 for porosity. Notably, only the flexural-strength model was employed to determine the hot-pressing parameters. The porosity quadratic model displayed significant lack of fit (*p* = 0.0046), attributed to extremely small pure experimental error; therefore, the porosity model is reported purely for descriptive purposes and was not utilized for process optimization. The target fiber content of 64.60 wt% was the value that maximized ILSS in a separate fiber-content optimization (S2 Tables S4 and S5). The laminate thickness was 2.0 ± 0.1 mm. Test specimens were cut on a Charly 2U universal prototype machine.

### 2.5. Characterization

FTIR (Nicolet iS50, ATR mode, Thermo Fisher Scientific, Madison, WI, USA) was recorded with 16 scans at 2 cm^−1^ resolution from 400 to 4000 cm^−1^. XPS (Axis Supra+, Kratos Analytical Ltd., Manchester, UK; Shimadzu Corporation, Kyoto, Japan) used a monochromated Al Kα source (1486.6 eV); high-resolution C 1s spectra were calibrated at 284.8 eV and deconvoluted with an asymmetric line shape (A(0.4, 0.5, 0) GL in XPS Peak 4.1) for the sp^2^ component, with the FWHM of all sub-peaks constrained to within ±0.2 eV. SEM (Hitachi SU8230, Hitachi High-Tech Corporation, Tokyo, Japan) at 3.0 kV was used for surface and fractographic morphology. Contact angles of water (γ_lv = 72.8 mN·m^−1^, γ_lv^d^ = 21.8, γ_lv^p^ = 51.0) and diiodomethane (γ_lv = 50.8, γ_lv^d^ = 49.5, γ_lv^p^ = 1.3) were measured on a Contact angles (DSA25, Krüss GmbH, Hamburg, Germany) at 25 ± 0.1 °C, with five replicates per specimen; the Owens–Wendt–Rabel–Kaelble (OWRK) method [23,24] was used to compute surface energy. Tensile, flexural, and short-beam shear tests were performed on MTS equipment. Tensile strength was measured per GB/T 1447-2023 [25] at 10 mm·min^−1^ on dumbbell-shaped specimens. Flexural strength and modulus were measured by three-point bending per GB/T 1449-2005 [26] at 2 mm·min^−1^. ILSS was measured by short-beam three-point bending per ISO 14130 [27] at 2 mm·min^−1^, span-to-thickness ratio 5:1; only specimens failing by interlaminar shear were accepted. Laminate density was measured by the water-displacement Archimedes method per ASTM D792-20 [28] at 23 ± 0.5 °C on three replicate specimens per group, and void content was calculated as X_v = (1 − ρ_meas/ρ_theory) × 100% with ρ_theory = 1/(w_CF/ρ_CF + (1 − w_CF)/ρ_PEEK), where w_CF is the mass fraction of carbon fiber, with ρ_CF = 1.78 g·cm^−3^ (T700) and ρ_PEEK = 1.30 g·cm^−3^. For the present 64.60 wt% CF/33.03 wt% PEEK composition, this gives ρ_theory = 1.597 g·cm^−3^. Each data point in Table 2 is the mean of at least five valid specimens cut from a single representative laminate per formulation, reported with standard deviation (SD). The present dataset involves two fundamentally different sampling designs, which require different statistical treatments and which are reported in two different sections of the Supplementary Information. Two distinct experimental designs were adopted in this work. The hot-pressing optimization study (S1 Tables S2 and S3) used a Box–Behnken response surface design with 15 independently fabricated laminates; the quadratic regression ANOVA is therefore valid. In contrast, the formulation comparison (S5 Table S8) adopted a within-plate design: each formulation corresponds to one laminate, and the five specimens per group are technical replicates cut from that single plate. These technical replicates violate the independence assumption for one-way ANOVA. Thus, we report Cohen’s d effect sizes to quantify group differences relative to within-group standard deviation. Our conclusions rely on the magnitude of observed effects and consistent trends across filler concentrations, not on individual *p*-values from S5 Table S8.

Density and porosity calculated via the Archimedes water-displacement method for selected specimens are provided in Supplementary Table S7. For the measured samples, porosity values stay low within a narrow window of 0.76–1.16% across different sizing formulations, with a maximum inter-group difference of only ~0.40%. This modest variation in void content is insufficient to explain the large increases in ILSS and flexural properties. Accordingly, the substantial mechanical improvements are predominantly attributed to interfacial modification by SPEEK-based composite sizing. Porosity data are currently unavailable for CF-B, CF-GO-1, and CF-CNT-3.

Statistical treatment of the present data. The reported standard deviations in Table 2 represent the variability among the five specimens cut from a single representative laminate per formulation, i.e., the within-laminate variability. They are used to assess the repeatability of the mechanical tests and the data scatter, not for formal statistical inference of population differences between formulations. Conclusions regarding material performance in the present work are based on (i) the magnitude and consistency of the observed mean value trends across the three nanofiller concentrations; they do not rest on any single *p*-value as a hypothesis-testing result. A confirmatory study with n = 3–5 independent plates per group and nested ANOVA with plate as a random factor is the subject of ongoing work.

## 3. Results and Discussion

### 3.1. Surface Chemistry

**Figure 2:**
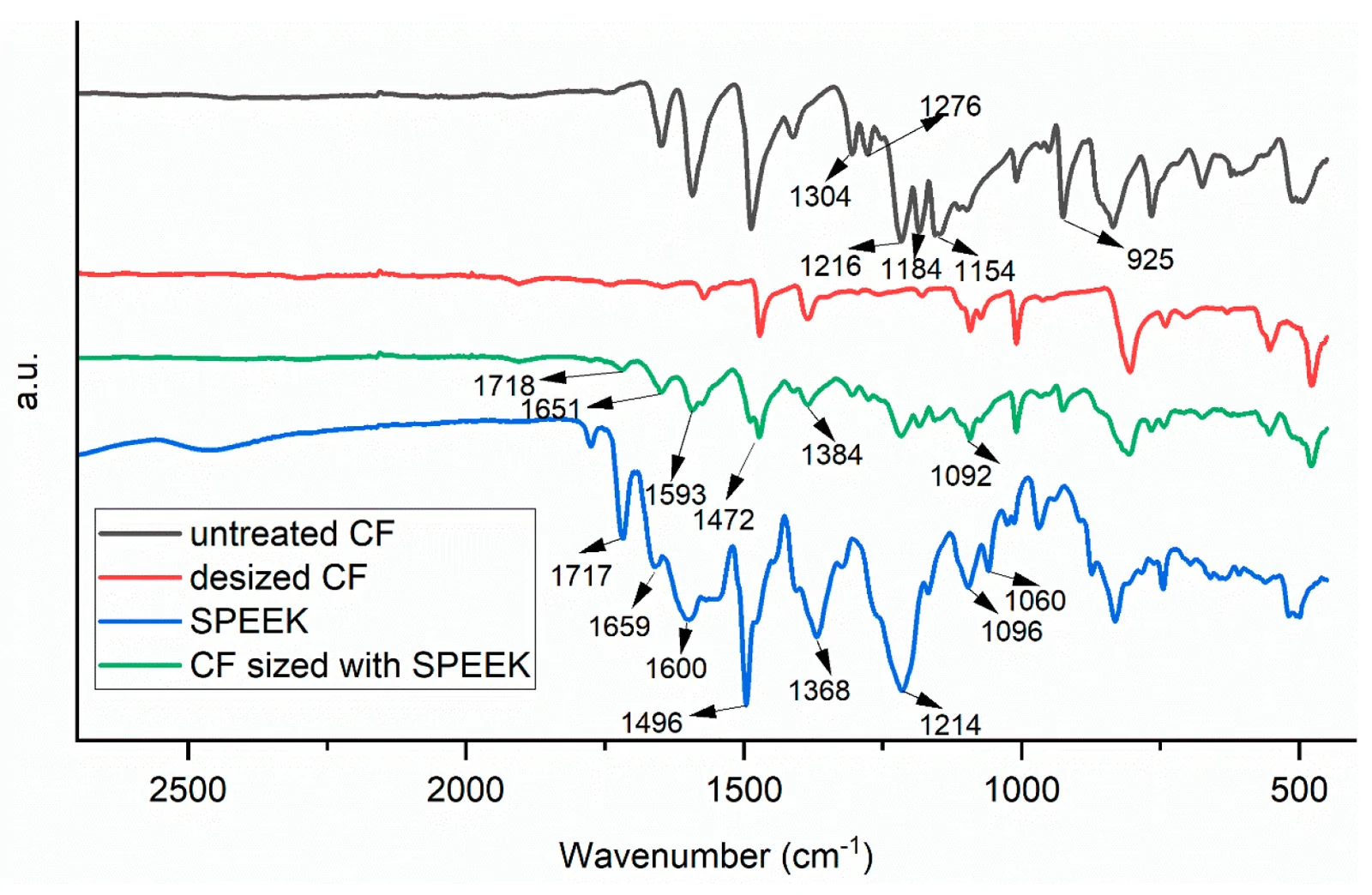
FTIR spectra of CF-0, CF-B, pure SPEEK powder, and CF-3. Peak assignments: (i) 1080 cm^−1^ S=O stretch of –SO_3_H; (ii) 1020 cm^−1^ C–S stretch; (iii) 1659 cm^−1^ C=O of PEEK; (iv) 1600/1496 cm^−1^ aromatic skeletal stretch; (v) 1368 cm^−1^ aromatic C–H bending.

The FTIR spectra of CF-B, pure SPEEK, and the SPEEK-sized CF (CF-3) are shown in Figure 2. After acetone desizing, the characteristic epoxy peak at 925 cm^−1^ and the C–O/C–C/ether peaks at 1154, 1184, 1216, 1276, and 1304 cm^−1^ were significantly weakened, confirming removal of the commercial sizing. Following SPEEK deposition, all the characteristic SPEEK peaks appeared in CF-3, including the S=O stretch of –SO_3_H at 1080 cm^−1^, the C–S stretch at 1020 cm^−1^, the aromatic C–H bending at 1368 cm^−1^, the carbonyl C=O of the PEEK backbone at 1659 cm^−1^, and the aromatic skeletal stretches at 1600 and 1496 cm^−1^. The persistence of these bands confirms that the molecular structure of SPEEK survived the sizing process and that the polar –SO_3_H groups remained at the interface.

**Figure 3:**
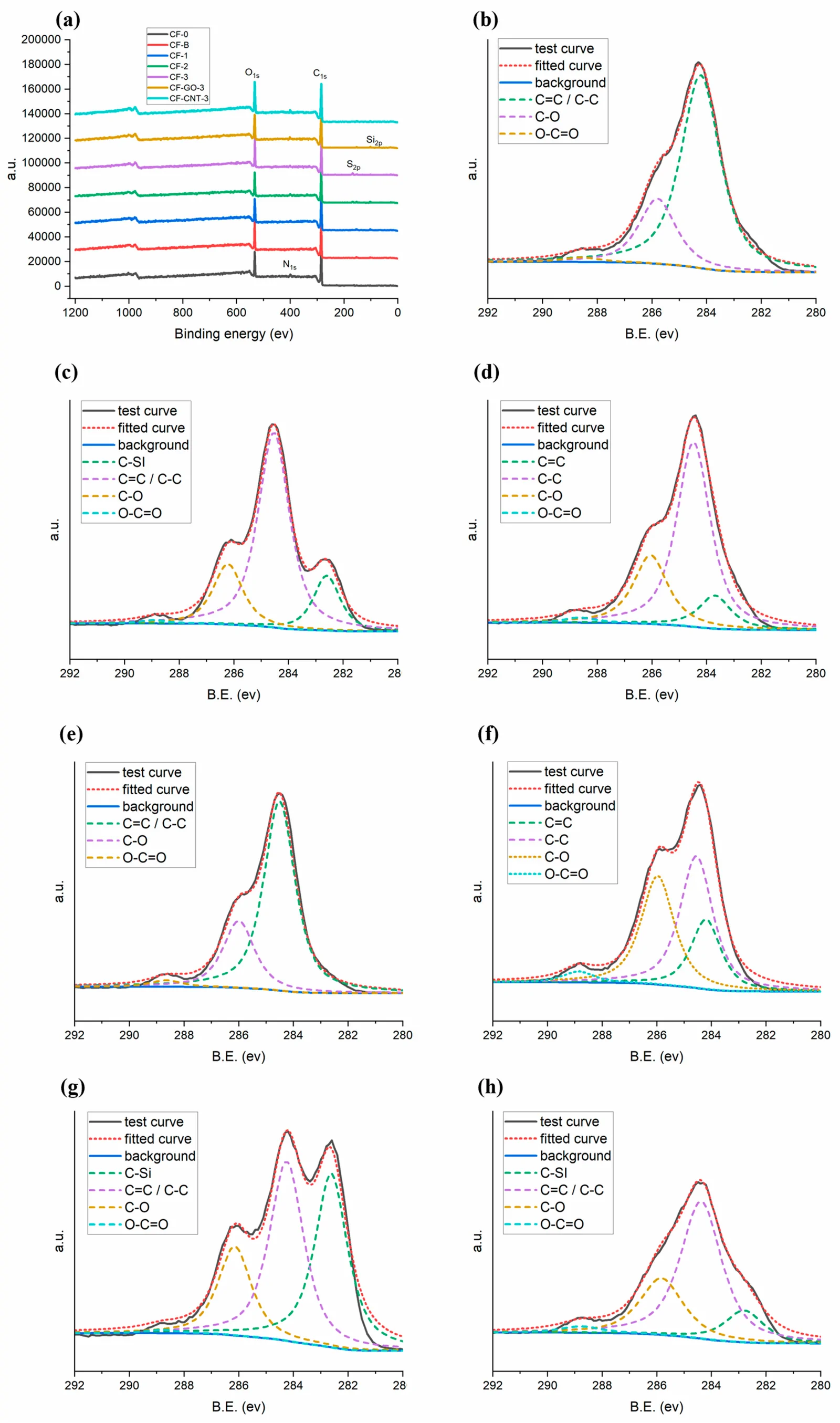
(**a**) XPS wide-scan spectra of CF-0, CF-B, CF-1, CF-2, CF-3, CF-GO-3, and CF-CNT-3 and (**b**–**h**) high-resolution C 1s spectra of the seven CF specimens. Note: 283 eV binding energy is consistent with SiC and is more parsimoniously explained as a fit artefact than as a chemical C–Si bond on the SPEEK-coated fiber surface.

The XPS wide-scan spectra (Figure 3) showed that SPEEK deposition progressively increased the O 1s intensity and reduced the C 1s intensity; the O content reached 41–42 at.% in CF-GO-3 and CF-CNT-3, consistent with the co-deposition of oxygen-rich GO and the SPEEK shell on the CNT. The C 1s deconvolution showed that the C–O/C=O fraction increased from 18.87% (CF-B) to 34.46% (CF-3) after SPEEK deposition. For the composite-sized samples, the C–O/C=O fraction is influenced by two competing effects: (a) the additional oxygen-bearing groups of GO and the SPEEK shell on CNT tend to raise the C–O/C=O fraction; (b) the sp^2^-rich carbon skeleton of GO and CNT, which carries a smaller polar fraction than the SPEEK overlayer, dilutes the polar contribution to the C 1s envelope. The present asymmetric-line-shape fit gave 19.44% (CF-GO-3) and 25.50% (CF-CNT-3); the lower value for CF-GO-3 relative to CF-3 is therefore attributed to sp^2^-carbon dilution rather than to a loss of polar groups.

Quantitative balance of polar vs. sp^2^ contributions. The GO loading of 2.0 g·L^−1^ in a 0.010 g·mL^−1^ SPEEK sizing corresponds to a GO/(GO + SPEEK) nominal mass ratio of 2.0/(2.0 + 10) = 16.7 wt%. The polar oxygen-bearing groups of GO are therefore a minority of the total carbon in the C 1s envelope, whereas the sp^2^-rich skeleton of GO contributes a large non-polar fraction. Combining (a) the addition of ≈16.7 wt% GO carbon and (b) the dilution of the polar SPEEK overlayer by this non-polar sp^2^ carbon explains the decrease of the C–O/C=O fraction from 34.46% (CF-3) to 19.44% (CF-GO-3). The CNT system has the same nominal sp^2^/(SPEEK + sp^2^) mass ratio of 16.7 wt% (CNT at 2.0 g·L^−1^ over SPEEK at 10 g·L^−1^), but the higher carbon content of the CNT sidewall (no oxygen-bearing functional groups) means the C–O/C=O fraction in CF-CNT-3 decreases only to 25.50% rather than the 19.44% of CF-GO-3, consistent with a less pronounced polar-group contribution from the SPEEK overlayer per unit mass of sp^2^ carbon. We emphasize that these 16.7 wt% values are nominal solution concentrations; the actual deposited mass fractions on the fiber surface were not measured by TGA in the present study. It should also be noted that the nominal solution concentrations of GO and CNT (0.5, 1.0, 2.0 g·L^−1^) do not guarantee strictly equal deposited mass on the fiber surface. Even under identical solution composition, sonication, immersion, and drying conditions, differences in particle dimension, surface charge, and settling tendency may create small deviations in real deposited loading between GO and CNT groups. Direct TGA quantification of recovered sized fabric would be necessary to accurately determine the actual nanofiller mass fraction deposited onto the fiber.

S 2p detection limit. SPEEK with DS ≈ 0.78 has a nominal S mass fraction of ≈2.6 wt% and a corresponding atomic fraction in a pure SPEEK overlayer of ≈1.2 at.%. On a CF surface covered by a thin SPEEK overlayer (≈20–100 nm by SEM cross-section), the expected S signal at the XPS sampling depth (≈5–10 nm for Al Kα) is therefore on the order of 0.1–0.5 at.%. Under the present acquisition (monochromated Al Kα, 1486.6 eV; pass energy 80 eV; survey scan summed over five sweeps; charge correction to C 1s at 284.8 eV), the practical detection limit estimated from the survey noise floor is ≈0.3–0.5 at.%, so the S 2p peak at ≈168 eV is expected to be at or below the detection limit.

Surface vs. bulk sensitivity of the SPEEK retention evidence. The present XPS data should be interpreted as surface-sensitive (<10 nm sampling depth) evidence of the chemical state of the topmost fiber surface. The absence of a detectable S 2p signal at ≈168 eV (estimated detection limit 0.3–0.5 at.%) indicates that the SPEEK overlayer is either very thin (<5 nm) or only patchy on the topmost surface; the O 1s increase from CF-B to CF-3 and the corresponding C–O/C=O fraction increase (Section 3.1) are therefore attributed to a combination of the SPEEK overlayer, the inherent oxygen-containing groups on the CF surface (e.g., –OH, –COOH from the carbon fiber manufacturing process), and possible air-borne hydrocarbon contamination. The O 1s data are taken as consistent with, but not as direct proof of, SPEEK deposition. The more direct evidence for the retention of the SPEEK molecular structure (–SO_3_H, ether, carbonyl, aromatic backbone) after the hot-pressing step comes from the FTIR spectrum, which has a much larger sampling depth (≈0.5–2 μm) and clearly shows the S=O/C–S/aromatic bands of SPEEK on the CF surface.

### 3.2. Surface Microstructures

**Figure 4:**
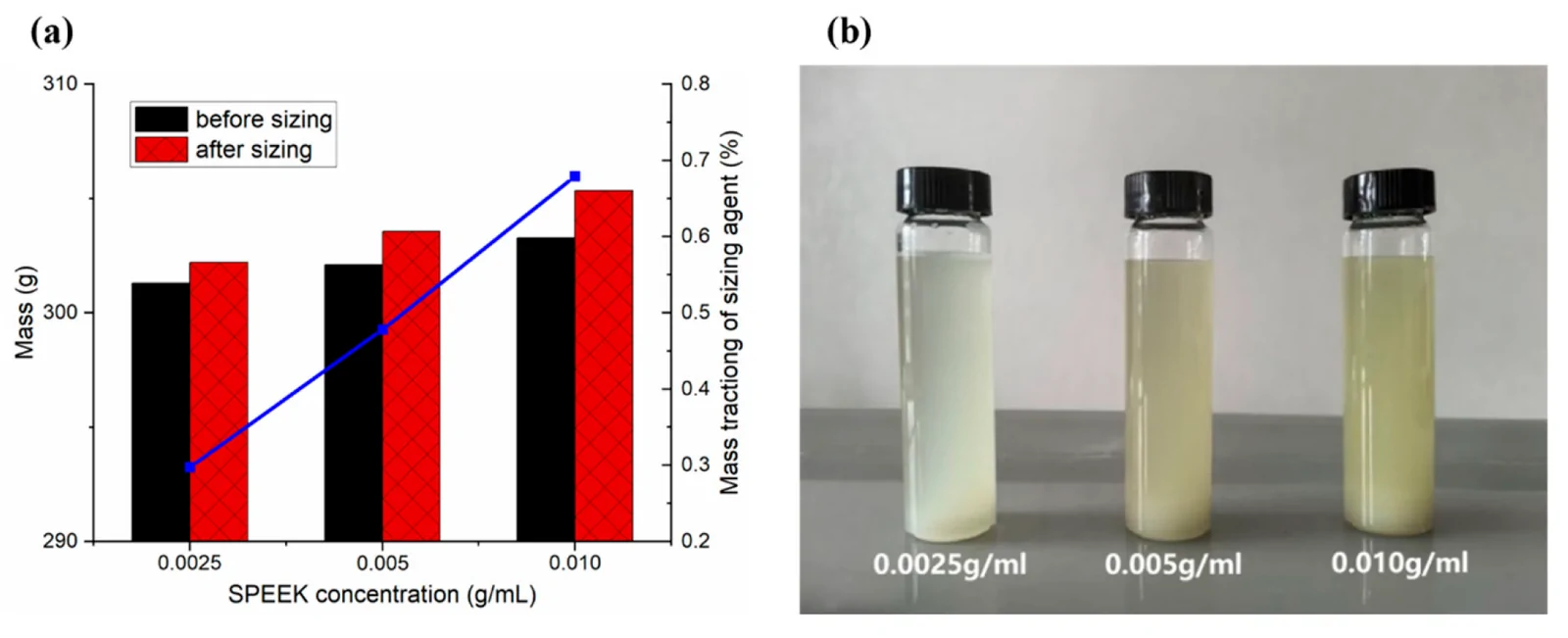
(**a**) Mass fraction of SPEEK adsorbed on the CF surface as a function of SPEEK concentration. (**b**) Appearance of the SPEEK solutions at 0, 0.0025, 0.005, and 0.010 g·mL^−1^.

**Figure 5:**
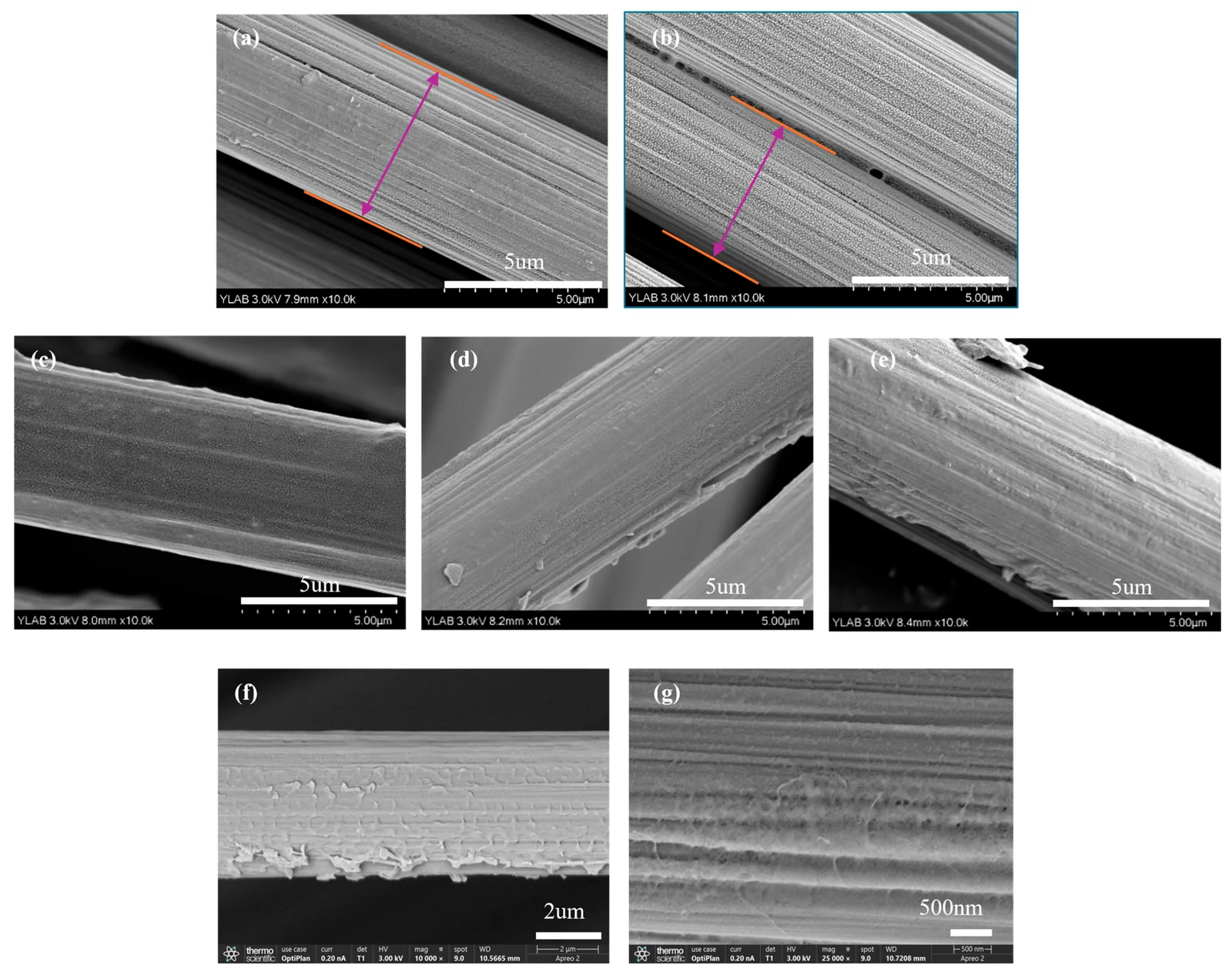
SEM images of (**a**) CF-0, (**b**) CF-B, (**c**) CF-1, (**d**) CF-2, (**e**) CF-3, (**f**) CF-GO-3, (**g**) CF-CNT-3.

The mass fraction of SPEEK adsorbed on the CF surface increased monotonically with the bulk SPEEK concentration (Figure 4a), and the sizing solution became visibly darker at higher concentrations (Figure 4b). The corresponding SEM morphologies are shown in Figure 5. The precursor CF (Figure 5a) exhibits a smooth surface with shallow longitudinal grooves, masked by the commercial epoxy sizing. After acetone desizing (Figure 5b), the grooves become more pronounced. After SPEEK sizing (Figure 5c–e), a uniform polymer film forms on the fiber surface; the film thickness grows from tens of nanometers at CF-1 to more than 100 nm at CF-3. The GO/SPEEK treatment (Figure 5f) increases the surface roughness, with GO sheets uniformly covering and partially embedding into the SPEEK matrix. The CNT/SPEEK treatment (Figure 5g) produces a distinct nano-network structure, with CNTs interpenetrating the fiber grooves. This 3D network is expected to provide stronger mechanical interlocking than the 2D GO sheet geometry.

### 3.3. Surface Wettability

**Figure 6:**
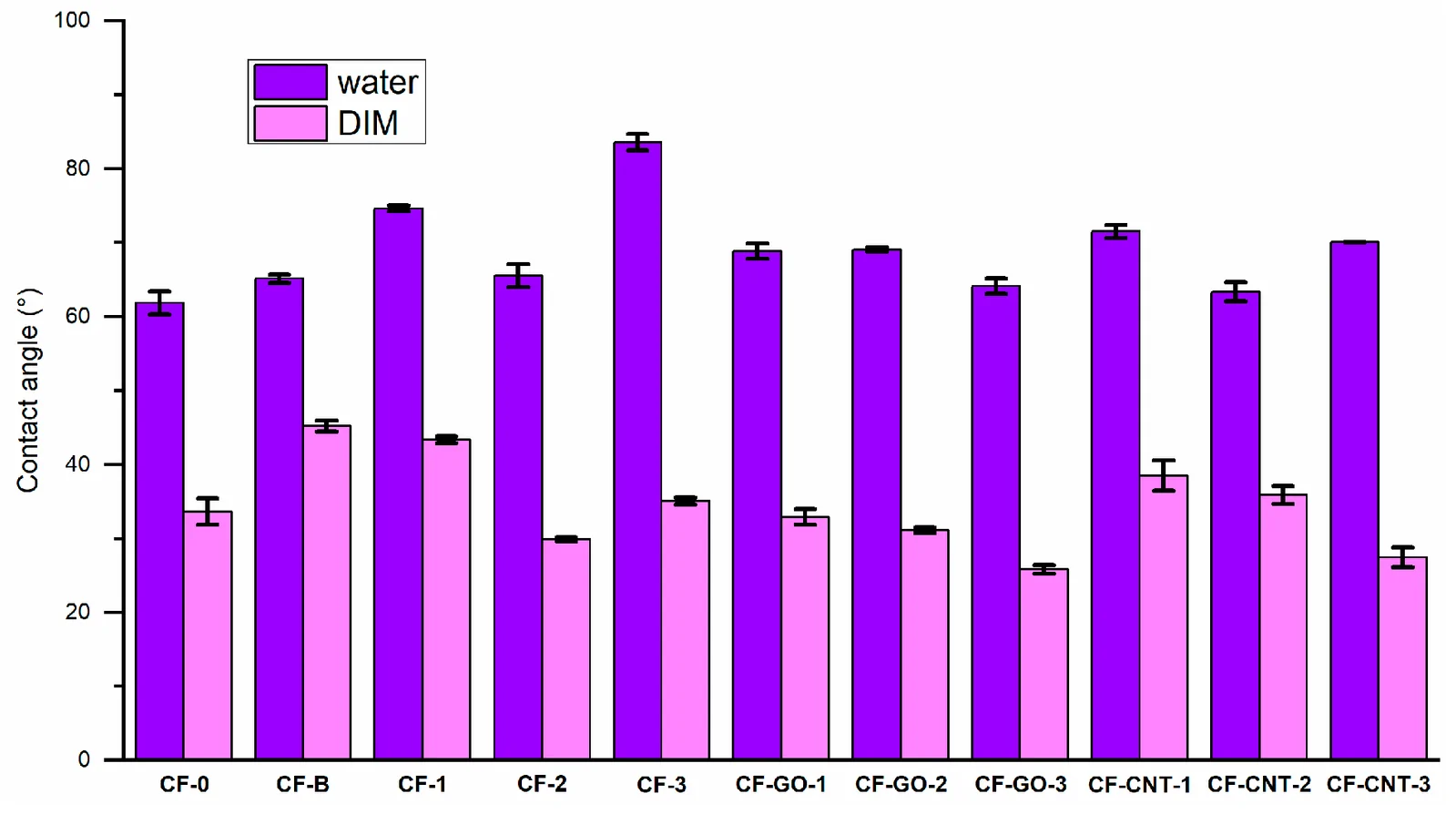
Water contact angle of the 11 CF/PEEK specimens.

**Figure 7:**
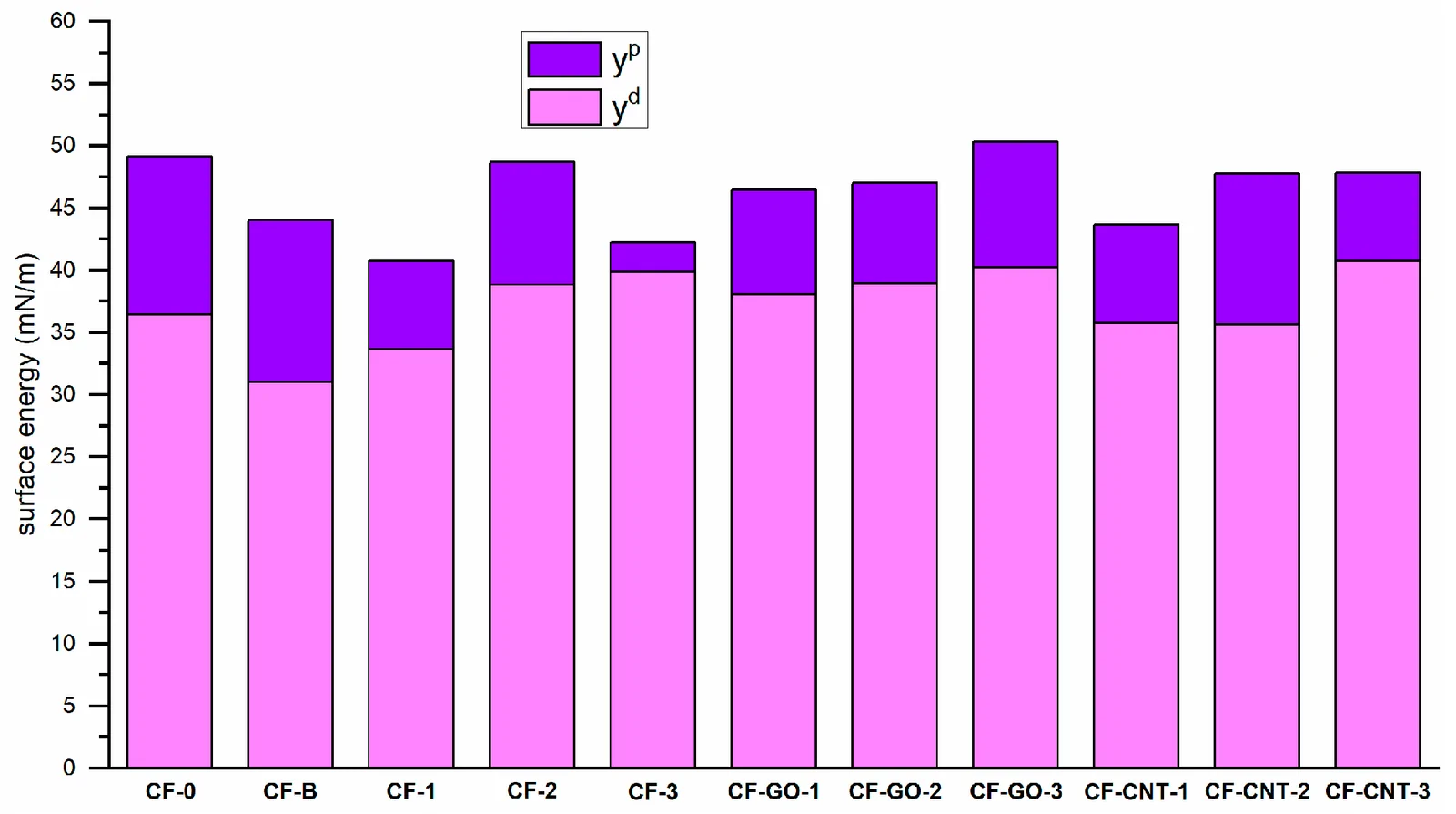
Total surface energy γ_s (bars) and its polar component γ^p^ (markers) of the 11 specimens.

Contact angles and OWRK surface energies of the 11 specimens are summarized in Figure 6 and Figure 7. After acetone desizing, CF-B had a water contact angle of ≈65° and a total surface energy γ_s of 40.75 mN·m^−1^. The SPEEK-only series showed a non-monotonic concentration dependence: γ_s rose to 48.68 mN·m^−1^ at CF-2 (0.005 g·mL^−1^) and then fell to 42.24 mN·m^−1^ at CF-3 (0.010 g·mL^−1^). The water contact angle of CF-3 (83.58°) is, surprisingly, higher than that of CF-1 (74.63°), even though the SPEEK film at CF-3 is thicker, and SPEEK is nominally water-soluble. We attribute this to surface reconstruction of the amphiphilic SPEEK film during the slow drying step (60 °C, ambient atmosphere): although 60 °C is below the PEEK Tg (≈143 °C), the polar–nonpolar segregation between the polar –SO_3_H groups and the nonpolar PEEK main chain during the early stage of water evaporation buries the polar groups sub-surface and presents a more hydrophobic PEEK-rich surface to the probe liquid. This interpretation is supported by the very low polar component γ^p^ of CF-3 (2.38 mN·m^−1^) and the high dispersive component γ^d^ (39.86 mN·m^−1^).

The composite sizings raised γ_s monotonically up to 50.34 mN·m^−1^ at CF-GO-3, with CF-CNT-3 slightly lower (47.83 mN·m^−1^). The polar component γ^p^, not the dispersive component γ^d^ (31.0–40.7 mN·m^−1^), provided the dominant contribution to the total surface energy variation. The fact that CF-CNT-3 has a lower γ_s than CF-GO-3 yet delivers higher ILSS and tensile strength (Section 3.3) indicates that the equilibrium wettability of the sized fiber is not the sole predictor of interfacial adhesion in the consolidated laminate: other factors, including melt impregnation during hot-pressing, mechanical interlocking by the CNT network, and residual thermal stresses, contribute significantly.

### 3.4. Mechanical Properties

**Figure 8:**
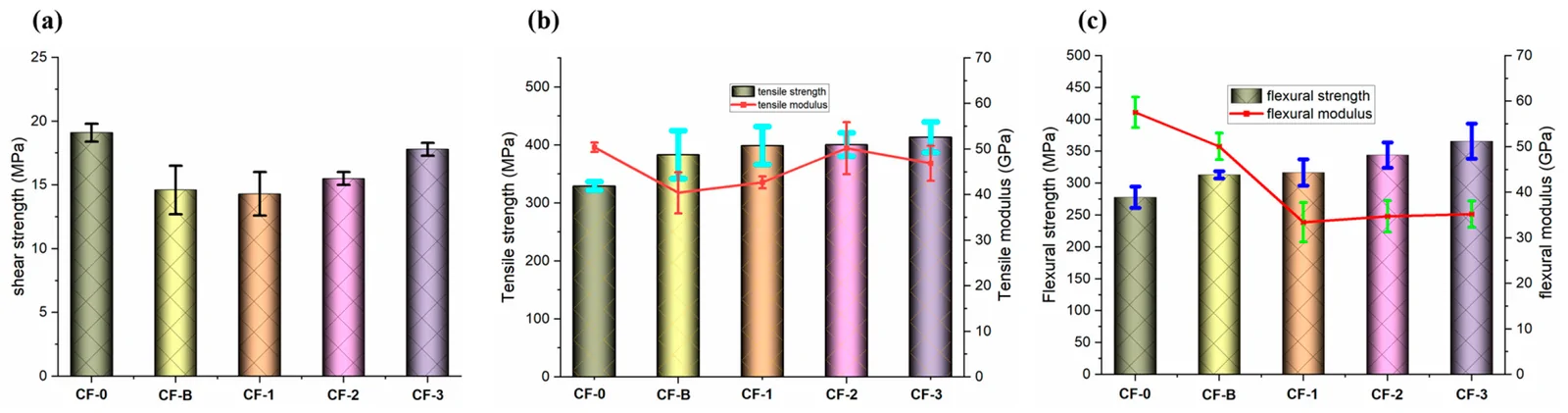
Mechanical properties of the SPEEK-only series: (**a**) ILSS, (**b**) tensile, (**c**) flexural.

**Figure 9:**
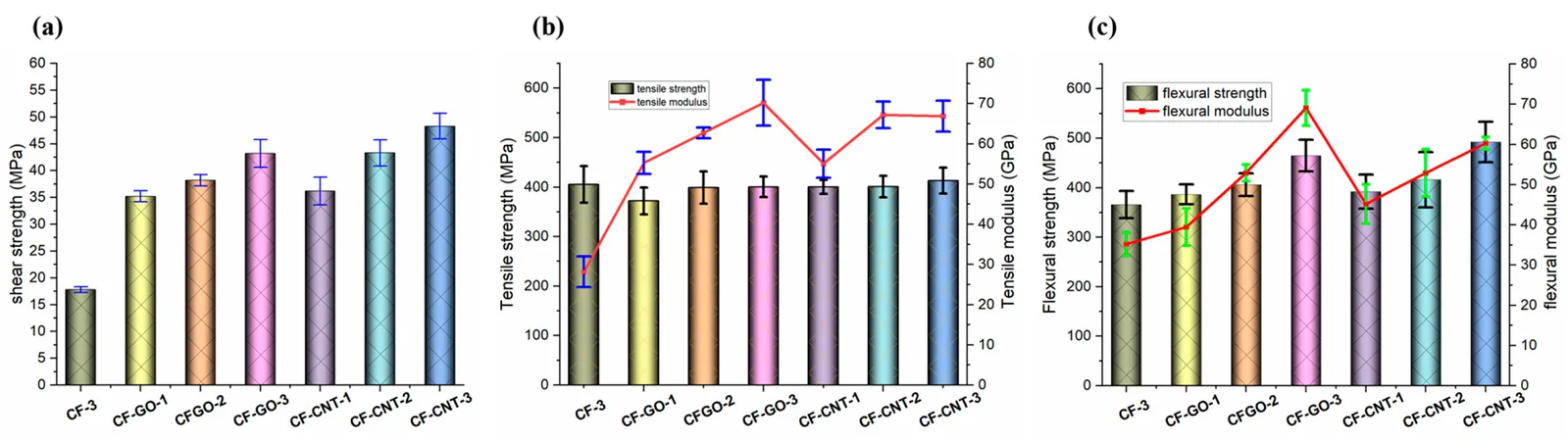
Mechanical properties of the SPEEK-only, GO/SPEEK, and CNT/SPEEK CF/PEEK laminates. (**a**) ILSS; (**b**) tensile strength and tensile modulus; (**c**) flexural strength and flexural modulus.

The ILSS, tensile, and flexural data of all 11 specimens are summarized in Table 2 and Figure 8 and Figure 9. For the SPEEK-only series, ILSS rose monotonically from 14.3 to 17.8 MPa as the SPEEK concentration increased from 0.0025 to 0.010 g·mL^−1^, a +22% gain over the desized CF-B (14.6 MPa). The tensile strength rose from 398.9 to 413.3 MPa (+8% over CF-B), and the flexural strength rose from 316.8 to 365.9 MPa (+17% over CF-B). The standard deviation decreased with increasing SPEEK concentration (ILSS SD from 1.9 to 0.5 MPa), indicating improved interfacial mechanical stability. The tensile moduli of the three baselines establish the contribution of each step: CF-0 (50.4 ± 1.06 GPa, commercial epoxy-sized) > CF-3 (46.9 ± 3.86 GPa, SPEEK-sized) > CF-B (40.4 ± 4.53 GPa, acetone-desized), i.e., acetone desizing reduces the tensile modulus by ≈10.0 GPa relative to the commercial baseline, and the SPEEK sizing recovers ≈ 6.5 GPa of this loss. The fact that CF-3 (46.9 GPa) remains below CF-0 (50.4 GPa) by ≈3.5 GPa despite the SPEEK being tailored for the CF/PEEK system is not a sizing failure but a fundamental property contrast: SPEEK is a linear thermoplastic (T_g ≈ 140–150 °C, E ≈ 1–2 GPa), whereas the commercial epoxy is a crosslinked thermoset (E ≈ 3–4 GPa), and the SPEEK deposit at 0.010 g·mL^−1^ is also thicker (a few tens of nm) than the sub-micron commercial epoxy layer. Acetone desizing further damages the rigid surface layer of the as-received CF in a separate characterization on the unsized CF bundle, so that part of the 10.0 GPa loss is not recoverable by any polymer re-coating. The same ranking is observed for the flexural moduli: CF-0 (57.6 ± 3.37 GPa) > CF-B (50.1 ± 2.93 GPa) > CF-3 (35.2 ± 2.89 GPa); here the SPEEK sizing does not recover the flexural-modulus loss, which is consistent with the soft SPEEK overlayer being more compliant than the rigid CF in the bending direction.

This apparent divergence between flexural modulus and flexural strength among baseline samples reflects competing interphase effects. The relatively compliant SPEEK polymer interlayer of CF-3 lowers the macroscopic flexural modulus relative to CF-0 and CF-B. Nevertheless, the improved fiber-matrix interfacial bonding introduced by SPEEK sizing inhibits premature interfacial debonding during bending, which yields higher flexural strength. Consequently, a softer interphase can improve failure strength by suppressing debond-driven fracture, even when it negatively influences laminate stiffness.

For the GO system, ILSS rose monotonically with concentration: 35.2, 38.2, and 43.2 MPa at 0.5, 1.0, and 2.0 g·L^−1^, equivalent to +141%, +163%, and +196% over the desized baseline. For the CNT system, ILSS rose from 36.2 to 43.3 to 48.3 MPa (+148%, +197%, and +231% over CF-B). At every concentration, the CNT system outperformed the GO system, and the difference at 2.0 g·L^−1^ (5.1 MPa) was approximately twice the larger of the two within-group standard deviations (2.39 MPa for CF-CNT-3 and 2.58 MPa for CF-GO-3, Table 2). The present experimental data show that CF-CNT-3 (48.3 MPa) exhibited a higher mean ILSS than CF-GO-3 (43.2 MPa) under the present experimental conditions. Two factors contribute: (i) the 1D tube geometry of CNT, with its high aspect ratio and interpenetrating network, provides a more effective load-bridging path across the interlaminar plane than the 2D face-to-face contact of GO, and (ii) the GO lamellae, while providing good in-plane coverage, cannot bridge across fiber/matrix gaps as effectively as elongated tubes. The data scatter also differs: GO’s SD increases from 1.04 to 2.58 MPa as concentration rises, indicating that uniform GO dispersion becomes more challenging at higher loading, while CNT’s SD is more stable (2.39–2.59 MPa), reflecting the higher entanglement tolerance of the 1D network.

The tensile data showed the same trend: GO rose from 448.0 to 509.0 to 586.4 MPa, and CNT rose from 469.2 to 532.4 to 642.4 MPa at 0.5, 1.0, and 2.0 g·L^−1^, respectively. The present experimental data show that the 2.0 g·L^−1^ CNT formulation exhibited a higher mean tensile strength (642.4 MPa) than the GO counterpart (586.4 MPa, a 56 MPa or 9.6% difference) under the present experimental conditions; the within-laminate SD (23.6 and 20.5 MPa, respectively) is much smaller than this between-formulation difference, so the mean trend is internally consistent. A different trend emerged for the tensile modulus: GO rose monotonically from 47.9 GPa (0.5 g·L^−1^) to 67.1 GPa (1.0 g·L^−1^) to 81.4 GPa (2.0 g·L^−1^), while CNT followed a non-monotonic pattern (51.7 → 87.1 → 82.6 GPa). The apparent decrease from 87.1 ± 5.69 GPa at 1.0 g·L^−1^ to 82.6 ± 1.34 GPa at 2.0 g·L^−1^ lies within the within-laminate experimental scatter. Accordingly, these data indicate a modulus plateau across the 1.0–2.0 g·L^−1^ concentration window, rather than a well-defined true optimum. Although local CNT entanglement may commence at loadings above 1.0 g·L^−1^, independent multi-plate replicates would be required to confirm whether a genuine performance maximum exists. The complete baseline chain is CF-0 (50.4 GPa) < CF-GO-3 (81.4 GPa) and CF-0 < CF-CNT-3 (82.6 GPa), giving +62% and +64% gains over the commercial epoxy baseline; the chain CF-3 (46.9 GPa) < CF-GO-3 (81.4 GPa) and CF-3 < CF-CNT-3 (82.6 GPa) gives +74% and +76% gains over the SPEEK-only baseline. The literature reports GO sizing loadings up to 5.0 g·L^−1^ [12] and CNT loadings up to 3.0 g·L^−1^ [16] in analogous aqueous sizing systems; the monotonic enhancement observed for the GO system in the present 0.5–2.0 g·L^−1^ range suggests that 2.0 g·L^−1^ is below the GO saturation threshold, while the CNT system already shows a modulus plateau in the same range. A direct quantitative comparison is complicated by differences in sizing matrix chemistry and fiber architecture, and the present data do not exclude a higher optimal concentration for either system.

Both GO and CNT systems showed monotonic concentration dependence in flexural strength, with CNT consistently higher: GO gave 386.6, 406.1, and 465.0 MPa, and CNT gave 392.2, 416.0, and 492.3 MPa at 0.5, 1.0, and 2.0 g·L^−1^. The flexural modulus, however, showed a different pattern at the highest concentration: the CF-GO-3 mean (69.1 GPa) exceeded the CF-CNT-3 mean (60.3 GPa) by 8.8 GPa under the present experimental conditions. This mean difference is large relative to the within-laminate SD of the two formulations (4.40 GPa for CF-GO-3 and 1.56 GPa for CF-CNT-3). The complete baseline chain is CF-0 (57.6 GPa) > CF-B (50.1 GPa) > CF-3 (35.2 GPa) for the three controls, and CF-GO-3 (69.1 GPa) > CF-3 (35.2 GPa) gives a +96% gain over the SPEEK-only baseline and a +20% gain over the commercial epoxy baseline; CF-CNT-3 (60.3 GPa) > CF-3 (35.2 GPa) gives a +71% gain over the SPEEK-only baseline and a +5% gain over the commercial epoxy baseline. This is the most prominent geometry-associated mean trend in the present dataset, and the most plausible interpretation is that the 2D GO lamella, oriented approximately parallel to the fiber surface and the beam axis, provides a stronger in-plane constraint against the longitudinal tensile/compressive deformation that dominates the flexural modulus in three-point bending, while the 1D CNT network, with its in-plane bridging ability, contributes less to the in-plane bending stiffness. The +96% flexural modulus gain at CF-GO-3 over the SPEEK-only baseline is substantial; alternative contributions to this enhancement, such as GO-induced interfacial crystallization of the PEEK matrix, cannot be fully excluded by the present dataset and are the subject of ongoing work.

The 1.0 g·L^−1^ CNT formulation deserves special attention as a balanced-economy option: it achieves a tensile modulus of 87.1 GPa (higher than the 2.0 g·L^−1^ GO system), respectable strength gains, and the lowest data scatter in tensile strength (SD = 8.8 MPa). The same formulation, however, showed a notably wider scatter in flexural strength (SD = 55.7 MPa, RSD = 13.4%) than the other CNT specimens (SD = 34.6–41.2 MPa, RSD = 8–9%) and the GO series (SD = 20.0–31.9 MPa, RSD = 5–7%). This suggests that 1.0 g·L^−1^ is at the upper edge of the uniform-dispersion window for CNT in the SPEEK matrix: local agglomerates start to form, but the bridging network is not yet fully interconnected, giving a high average but a wide sample-to-sample distribution. This interpretation is consistent with the tensile-modulus plateau in Section 3.3 and the surface-energy trend in Section 3.2 at the same concentration.

Comparison with the commercial epoxy sizing. The commercial epoxy-sized baseline (CF-0) has an ILSS of 19.1 ± 0.1 MPa and a flexural strength of 277.9 ± 16.8 MPa, both of which lie between the acetone-desized reference (CF-B: 14.6 MPa ILSS, 313.3 MPa flexural strength) and the SPEEK-only baseline (CF-3: 17.8 MPa ILSS, 365.9 MPa flexural strength). The 2.0 g·L^−1^ SPEEK/CNT system therefore raises the ILSS by +153% (48.3 vs. 19.1 MPa) and the flexural strength by +77% (492.3 vs. 277.9 MPa) over the commercial epoxy-sized reference, while the 2.0 g·L^−1^ SPEEK/GO system raises the ILSS by +126% and the flexural strength by +67%. The use of the acetone-desized reference (CF-B) as the primary baseline in the present work follows the standard practice of sizing studies, in which the acetone-desized fabric is the substrate onto which the SPEEK-based sizing is deposited; the CF-0→CF-B difference isolates the contribution of the SPEEK overlayer and the nanofillers, while the CF-B→CF-3/CF-GO-3/CF-CNT-3 differences isolate the contribution of the nanofillers.

### 3.5. Geometry-Associated Trends and Working Hypothesis

**Figure 10:**
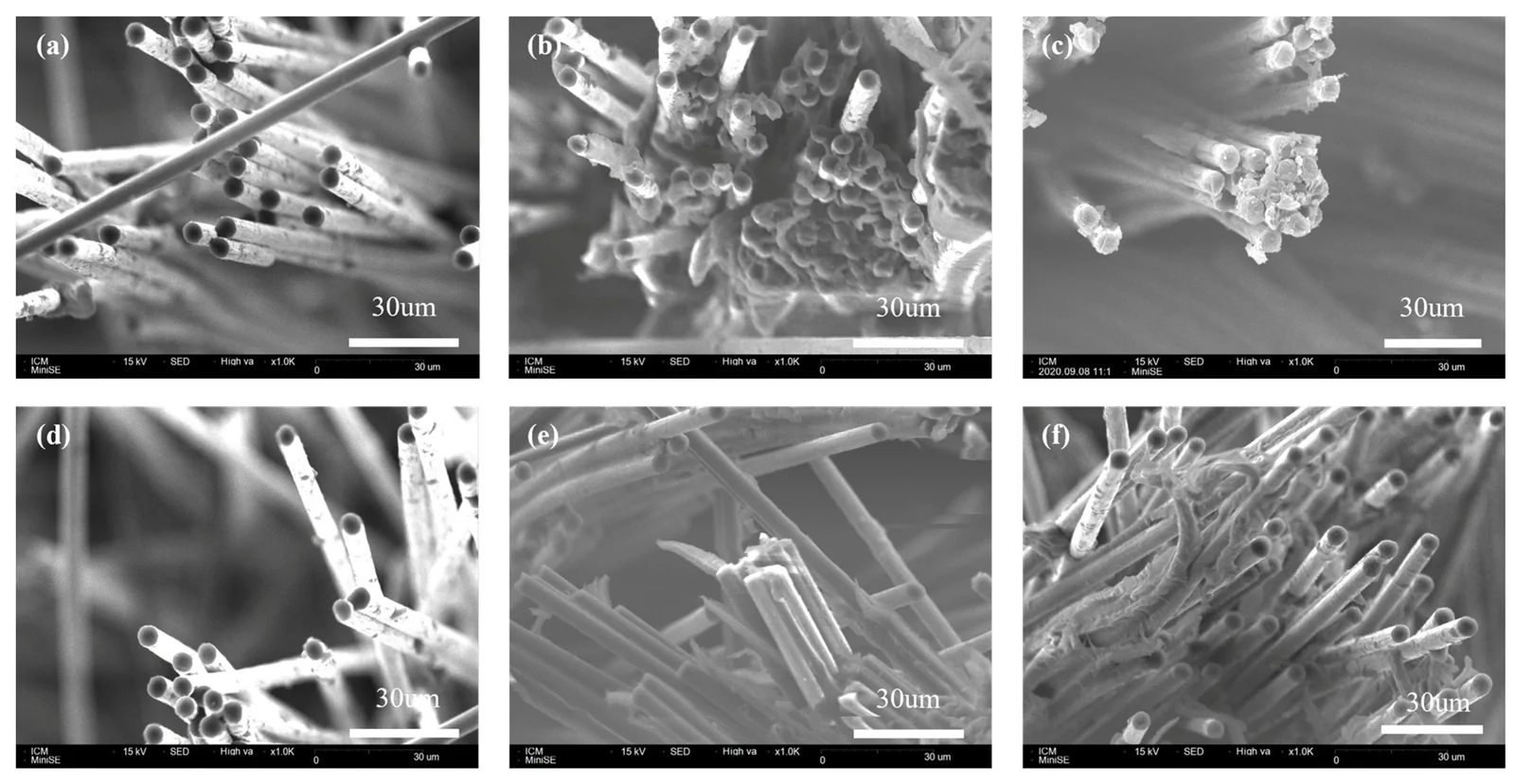
SEM fracture surfaces of (**a**) CF-B, (**b**) CF-3, (**c**) CF-GO-1, (**d**) CF-GO-2, (**e**) CF-GO-3, and (**f**) CF-CNT-3 after short-beam shear testing.

SEM fractographic analysis (Figure 10) showed a clear transition in failure mode with the addition of nanofillers. The desized specimen (CF-B) showed the classical “clean fiber pull-out + smooth matrix hole” pattern characteristic of pure adhesive interfacial debonding. The SPEEK-only specimen (CF-3) showed partial matrix retention on the fibers but with visible gaps at the interface. For nanofiller-modified specimens (CF-GO-3 and CF-CNT-3), three key fractographic markers indicate mixed cohesive-adhesive failure: abundant residual PEEK matrix adhering to carbon-fiber surfaces, shortened fiber pull-out lengths, and distinct matrix plastic tearing. This demonstrates that fracture does not propagate solely along the fiber-matrix boundary; portions of crack growth occur within the polymer matrix. Hence the failure evolves from pure adhesive interfacial debonding toward a mixed cohesive/adhesive failure mode, consistent with the mechanical trends described in Section 3.4.

Within the present single-plate screening dataset, the most plausible interpretation of the observed trends is that the nanoscale contact geometry between the rigid nanofiller and the CF surface is associated with the two mechanical signatures described in Section 3.3. GO is a 2D lamella with high in-plane rigidity and limited out-of-plane stiffness; it contacts the CF surface in a face-to-face manner, providing a large specific contact area (≈700–800 m^2^·g^−1^) and an effective in-plane constraint against beam-axis deformation. CNT is a 1D tube with a high aspect ratio (length 10–30 μm, diameter < 8 nm, aspect ratio ≈ 1000–4000) but a small specific contact area per unit mass; it contacts the CF surface in a line-to-face manner, providing a low contact area per unit mass but excellent in-plane bridging capability across the fiber–matrix interface. We present this geometry-based contact contrast as a working hypothesis consistent with the present data, not as a demonstrated mechanism, because (i) GO and CNT also differ in surface chemistry (C/O ratio, defect density, functional-group type), (ii) the present FTIR, XPS, and SEM evidence does not directly characterize the deposited nanofiller morphology on the CF surface, and (iii) the n = 5 within-plate sampling does not allow independent confirmation of the population-level geometric effect.

In addition to geometric disparities, GO and CNT fillers exhibit distinct chemical and surface characteristics that further regulate composite interfacial behavior. GO possesses a high oxygen content (up to ~50 at.%) with abundant hydroxyl and epoxy functional groups, which increase surface polarity and improve interfacial compatibility between nanofillers and the SPEEK interlayer/PEEK matrix. These chemically active sites promote filler-matrix intermolecular interactions and are conducive to the development of ordered transcrystalline PEEK at the interphase. By contrast, the as-received multi-walled CNTs have low oxygen content and relatively chemically inert side-wall surfaces, which yield comparatively weaker filler-matrix interactions and less pronounced matrix crystallization at the interface. This role of oxygen-containing groups can also be inferred by comparing GO and reduced GO variants: removal of surface oxygen moieties in reduced GO diminishes interfacial affinity towards the polymer matrix. Within our experimental system, the two-dimensional planar geometry of GO, enabling large-area interfacial constraint and stress redistribution, is considered the dominant factor responsible for elevated in-plane flexural modulus, while its abundant surface-oxygen chemistry acts as an important secondary auxiliary mechanism that further tailors interphase microstructure and load-transfer efficiency.

These two geometries translate into two distinct mechanical signatures. For in-plane stiffness (flexural modulus, dominated by tensile/compressive deformation along the beam axis in three-point bending), 2D face-to-face GO is the better choice (69.1 GPa at 2.0 g·L^−1^ vs. 60.3 GPa for CNT). For interlaminar-shear-strength and tensile-strength metrics, which require load transfer across or along the fiber–matrix interface, 1D line-to-face CNT is the better choice (48.3 MPa ILSS and 642.4 MPa tensile strength at 2.0 g·L^−1^ vs. 43.2 and 586.4 MPa for GO). The two systems also exhibit distinct concentration-dependence patterns: the GO system shows a monotonic, gradually saturating enhancement in the 0.5–2.0 g·L^−1^ range tested, while the CNT system shows a near-linear pattern for strength-related properties but a plateau in tensile modulus in the 1.0–2.0 g·L^−1^ range (87.1 → 82.6 GPa, within the within-laminate SD), consistent with the onset of local tube entanglement at higher loading. Saturation at higher concentrations (3.0–4.0 g·L^−1^) was not investigated.

It should be emphasized again that each formulation corresponds to only one independently hot-pressed laminate in this screening study, and n = 5 test specimens are technical replicates sectioned from that single plate. Therefore, we cannot completely rule out potential plate-to-plate manufacturing variability. We interpret the measured property differences as treatment-associated trends primarily because systematic, concentration-dependent mechanical evolutions are observed across three nanofiller concentrations, under strictly unified raw-material and hot-pressing conditions. These trends are presented as working hypotheses rather than validated general conclusions; multiple independent replicate plates per group are needed to further verify these observations.

Halpin–Tsai estimate of the nanofiller contribution. A semi-quantitative estimate of the contribution of the nanofillers to the laminate elastic modulus can be obtained from the Halpin–Tsai equation for a short-fiber composite, E = (3/8) E_L + (5/8) E_T, with E_L/E_m = (1 + 2(l/d)η_L V_f)/(1 − η_L V_f) and E_T/E_m = (1 + 2η_T V_f)/(1 − η_T V_f), where η_L = (E_f/E_m − 1)/(E_f/E_m + 2(l/d)) and η_T = (E_f/E_m − 1)/(E_f/E_m + 2). Using E_m = 3.6 GPa (amorphous PEEK), E_f(GO) = 250 GPa (in-plane), E_f(CNT) = 1000 GPa (axial), l/d(GO) = 5 × 10^3^ (in-plane), l/d(CNT) = 1.5 × 10^3^ (axial), and an interfacial layer thickness of 50 nm, the predicted contribution of the interfacial layer alone is ≈0.05 GPa to the laminate flexural modulus, which is two orders of magnitude smaller than the observed +38% gain (CF-GO-3 vs. CF-B). This semi-quantitative calculation helps to distinguish between direct nanofiller stiffening and geometry-driven indirect interphase modification, which underpins our core working hypothesis. This large discrepancy is a key piece of evidence: it rules out simple direct stiffening by rigid nanofillers as the primary source of modulus improvement. Instead, two indirect interphase mechanisms are proposed as plausible working hypotheses: (i) filler-induced restriction of polymer chain mobility within the interfacial region (the constraint effect of the 2D GO face-to-face contact on the in-plane deformation) and (ii) GO-promoted transcrystalline PEEK formation adjacent to carbon fibers. Direct experimental evidence confirming these interphase features is absent within the present dataset. Complementary characterizations including TEM, polarized optical microscopy, or DMA will be required in future work to validate chain-mobility confinement and transcrystalline microstructure. Both hypothesized interphase structural effects can substantially modify load transfer efficiency, which may account for the much larger experimentally measured modulus enhancement.

Within the limits of the present single-plate sample set, the geometry-associated trends suggest a practical starting point for interphase design in CF/thermoplastic composites: a GO-based composite sizing may be a reasonable starting point when the design driver is in-plane modulus, a CNT-based composite sizing may be a reasonable starting point when the design driver is interlaminar or tensile strength, and the 1.0 g·L^−1^ CNT formulation is attractive as a balanced-economy option. This design suggestion is presented as a working hypothesis to be tested on independently replicated laminates, not as a validated design rule, and it should be applied with the explicit caveat that the present n = 5 within-plate sampling does not support quantitative extrapolation to the CF/PEEK population.

## 4. Conclusions

This work investigates geometry-associated interphase modification of CF/PEEK laminates using SPEEK sizing functionalized with 1D CNT and 2D GO nanofillers. The Box–Behnken response-surface design enables identification of a suitable hot-pressing processing window based on flexural strength, with porosity data collected to confirm that void variation is minor and cannot explain the observed mechanical trends. Compared with the acetone-desized baseline, nanofiller-modified SPEEK interphases greatly boost interlaminar shear strength and tensile/flexural properties. SEM fractographic evidence shows a shift from adhesive interfacial debonding to mixed cohesive/adhesive failure, indicating stronger fiber–matrix interfacial integrity. The Halpin–Tsai model reveals that direct stiffening from nanofiller intrinsic stiffness is far too small to account for the large modulus gains. We hypothesize that the performance improvement originates from geometry-dependent interphase structural changes, including confinement of polymer chain mobility and modified transcrystallization near the carbon fiber surface.

This study identifies the benefit of nanofiller geometric variation as a viable strategy for thermoplastic composite interphase engineering. Importantly, we emphasize that the observed performance trends are correlated with nanofiller geometry rather than proven to be caused by geometry alone. GO and CNT differ inherently in surface chemistry, defect density, and dispersion state, which cannot be fully separated within this screening experiment. Further targeted interfacial characterization (TEM, POM, DMA) will be required to independently quantify geometric, chemical, and crystallographic contributions and validate the proposed interphase mechanisms.

## Figures and Tables

**Table 1:** Specimen codes and surface treatment conditions.

Sample	Treatment	SPEEK (g·mL^−1^)	Nanofiller	Nanofiller (g·L^−1^)
CF-0	Untreated (epoxy)	–	–	–
CF-B	Acetone-desized	–	–	–
CF-1	SPEEK	0.0025	–	–
CF-2	SPEEK	0.005	–	–
CF-3	SPEEK	0.010	–	–
CF-GO-1	SPEEK/GO	0.010	GO	0.5
CF-GO-2	SPEEK/GO	0.010	GO	1.0
CF-GO-3	SPEEK/GO	0.010	GO	2.0
CF-CNT-1	SPEEK/CNT	0.010	CNT	0.5
CF-CNT-2	SPEEK/CNT	0.010	CNT	1.0
CF-CNT-3	SPEEK/CNT	0.010	CNT	2.0

**Table 2:** Mechanical properties of the 11 CF/PEEK specimens.

Sample	ILSS (MPa)	Tensile Strength (MPa)	Tensile Modulus (GPa)	Flexural Strength (MPa)	Flexural Modulus (GPa)
CF-0	19.1 ± 0.1	329.2 ± 7.2	50.4 ± 1.06	277.9 ± 16.8	57.6 ± 3.37
CF-B	14.6 ± 1.9	383.2 ± 41.2	40.4 ± 4.53	313.3 ± 5.6	50.1 ± 2.93
CF-1	14.3 ± 1.7	398.9 ± 32.6	42.7 ± 1.31	316.8 ± 20.8	33.4 ± 4.32
CF-2	15.5 ± 0.5	400.6 ± 20.5	50.2 ± 5.73	344.1 ± 20.1	34.7 ± 3.44
CF-3	17.8 ± 0.5	413.3 ± 26.2	46.9 ± 3.86	365.9 ± 27.6	35.2 ± 2.89
CF-GO-1	35.2 ± 1.04	448.0 ± 14.0	47.9 ± 5.74	386.6 ± 20.0	39.5 ± 4.67
CF-GO-2	38.2 ± 1.07	509.0 ± 27.5	67.1 ± 1.15	406.1 ± 22.9	52.9 ± 2.09
CF-GO-3	43.2 ± 2.58	586.4 ± 20.5	81.4 ± 3.05	465.0 ± 31.9	69.1 ± 4.40
CF-CNT-1	36.2 ± 2.59	469.2 ± 19.6	51.7 ± 2.46	392.2 ± 34.6	45.2 ± 4.86
CF-CNT-2	43.3 ± 2.45	532.4 ± 8.8	87.1 ± 5.69	416.0 ± 55.7	52.9 ± 5.89
CF-CNT-3	48.3 ± 2.39	642.4 ± 23.6	82.6 ± 1.34	492.3 ± 41.2	60.3 ± 1.56

## Data Availability

All raw/processed data generated in this study are available within the article and its Supplementary Information Files. No additional external data are available.

## Supplementary Materials

The following supporting information can be downloaded at https://www.mdpi.com/article/10.3390/polym18202565/s1, Figure S1: Box–Behnken response surface and contour plots of flexural strength (σ_f); Figure S2: Box–Behnken response surface and contour plots of porosity (X_v); Figure S3: Box–Behnken model diagnostic plots; Table S1: Box–Behnken 15-run experimental data for hot-pressing process optimization of CF/PEEK commingled yarn fabric laminates; Table S2: ANOVA for the flexural strength (σ_f) quadratic regression model of the Box–Behnken hot-pressing optimization; Table S3: ANOVA for the porosity (X_v) quadratic regression model of the Box–Behnken hot-pressing optimization; Table S4: Carbon fiber content, fiber volume fraction, theoretical and measured density, and void content for CF/PEEK commingled yarn fabric laminates; Table S5: Carbon fiber content vs. mechanical properties for CF/PEEK commingled yarn fabric laminates (n = 5); Table S6: FTIR peak assignments for PEEK and SPEEK; Table S7: Thermogravimetric characteristics of the eleven CF/PEEK specimens under N_2_; Table S8: Effect-size summary of the most contested comparisons in the GO and CNT series; Table S9: The fiber content and porosity of the CF/PEEK samples were determined by acid digestion method; Note S1: DS titration protocol for sulfonation-degree measurement of SPEEK. (Note S1).
