# Supplementary material for: Geometry-Associated Mechanical Trends of Graphene Oxide and Carbon Nanotube SPEEK Sizings in CF/PEEK Commingled Yarn Laminates
Source: Polymers (Basel). 2026 Oct 18;18(20):2565. doi: 10.3390/polym18202565 (PMC13709323; doi:10.3390/polym18202565)
Supplement: Supplementary file 1 [file polymers-18-02565-s001.zip › polymers-4552447-supplementary.pdf]

Supplementary Information

Geometry-Associated Mechanical Trends of Graphene Oxide and Carbon Nanotube SPEEK Sizings in CF/PEEK Commingled Yarn Laminates

Xuejiang Yu[1]*, Yousi Chen[2], Ai Lu[1]*

[1] *National Key Laboratory of Chemical Explosion Safety, Institute of Chemical Materials, China Academy of Engineering Physics (CAEP), Mianyang 621900, China*

[2] *School of Materials Science and Engineering, Dalian University of Technology, Dalian 116024, China*

* *Correspondence: 3845644@qq.com (X. Y.) Tel.: +86-816-2495183*

* *Correspondence: ai_lu@sina.com (A. L.) Tel.: +86-816-2484330*

This Supplementary Information contains: (S1) Box–Behnken 15-run data, ANOVA tables, model diagnostic plots (Figure S3), and 3D response surface / 2D contour plots of the flexural strength (Figure S1) and porosity (Figure S2) models; (S2) fiber content, theoretical density, and void content table (Table S4) and fiber content vs. mechanical properties table (Table S5); (S3) FTIR peak assignments for PEEK and SPEEK; (S4) TGA characteristics of the eleven CF/PEEK specimens (Table S7); (S5) descriptive statistics of the GO and CNT series (Table S8, effect-size Cohen's d summary; see caption for the caveat on within-plate sampling); (S6) Notes, including Note S1 (DS titration protocol) and Note S2 (rationale for the asymmetric XPS C 1s fit).

S1. Hot-Pressing Process Optimization (Box–Behnken Design)

Three factors (A: molding temperature; B: molding pressure; C: holding time) and two responses (flexural strength σ_f and void content X_v) were selected. Three levels of each factor: 330/350/370 °C for temperature, 1.0/2.0/3.0 MPa for pressure, 10/20/30 min for holding time. The center point (350 °C / 2.0 MPa / 20 min) was replicated three times. The 15-run data are listed in Table S1. The molding temperature was the most significant factor for the σ_f model ($P = 0.0002$), followed by holding time ($P = 0.0083$) and pressure; the temperature–pressure interaction was also significant for the porosity model ($P = 0.0044$). The quadratic regression model for σ_f achieved $R^2 = 0.9905$ and adjusted $R^2 = 0.9733$, with a non-significant lack-of-fit ($P = 0.2006$, indicating adequate fit). The quadratic model for X_v achieved $R^2 = 0.9817$ and adjusted $R^2 = 0.9489$; the lack-of-fit for X_v was significant ($P = 0.0046$), attributable to the very small pure error of the porosity data at the center point ($X_v = 0.77 \pm 0.02\%$, $n = 3$). Despite this, the predicted optimum (350 °C / 2.0 MPa / 20 min) matches the experimental verification within ± 2%, and the model is considered adequate for the present process-screening purpose. The recommended hot-pressing window, based on the present Box–Behnken model, is 345–355 °C / 1.5–2.5 MPa / 15–25 min. The 3D response surface and 2D contour plots of the flexural strength model are given in Figure S1, and the corresponding plots of the porosity model are given in Figure S2.

Run	A: T (°C)	B: P (MPa)	C: t (min)	σ_f (MPa)	X_v (%)	Type
1	350	1.0	30	264.6	1.03	Edge
2	370	3.0	20	250.8	2.14	Edge
3	370	1.0	20	250.6	1.38	Edge
4	330	2.0	10	203.4	3.25	Edge

5	350	2.0	20	279.5	0.75	Center
6	350	1.0	10	251.4	1.34	Edge
7	350	3.0	10	256.7	1.22	Edge
8	370	2.0	30	238.6	2.04	Edge
9	350	3.0	30	262.9	0.89	Edge
10	330	2.0	30	232.4	2.53	Edge
11	330	1.0	20	226.5	4.12	Edge
12	330	3.0	20	214.7	2.64	Edge
13	370	2.0	10	242.9	1.86	Edge
14	350	2.0	20	275.2	0.79	Center
15	350	2.0	20	277.9	0.77	Center

Table S1. Box–Behnken 15-run experimental data for hot-pressing process optimization of CF/PEEK commingled yarn fabric laminates. Center-point mean: σ_f = 277.5 ± 2.3 MPa, X_v = 0.77 ± 0.02% (n = 3). Predicted optimum: 353.3 °C / 1.9 MPa / 21.7 min.

Source	SS	df	MS	F	P
Model	7071.06	9	785.67	57.67	0.0002 ***
A–Temperature	1401.85	1	1401.85	102.90	0.0002 ***
B–Pressure	8.00	1	8.00	0.59	0.4781 n.s.
C–Time	243.10	1	243.10	17.84	0.0083 **
AB	36.00	1	36.00	2.64	0.1650 n.s.
AC	277.22	1	277.22	20.35	0.0063 **
BC	12.25	1	12.25	0.90	0.3866 n.s.
A^2	4713.50	1	4713.50	345.97	< 0.0001 ***
B^2	139.84	1	139.84	10.26	0.0239 *
C^2	575.00	1	575.00	42.21	0.0013 **
Residual	68.12	5	13.62		
Lack of fit	58.67	3	19.56	4.14	0.2006 n.s.
Cor Total	7139.18	14			

Table S2. ANOVA for the flexural strength (σ_f) quadratic regression model of the Box–Behnken hot-pressing optimization. This is a standard regression ANOVA based on the complete 15-run data set (Table S1) and the quadratic model; the F values, degrees of freedom, and p values are therefore inferential statistics for the regression model itself, not descriptive summaries of composite material properties. σ_f = 277.53 + 13.24 A − 1.00 B + 5.51 C + 3.00 AB − 8.33 AC − 1.75 BC − 35.73 A^2 − 6.15 B^2 − 12.48 C^2. R^2 = 0.9905, adjusted R^2 = 0.9733, CV = 1.49%. *** P < 0.001, ** P < 0.01, * P < 0.05, n.s. P > 0.05.

Source	SS	df	MS	F	P
Model	13.97	9	1.55	29.87	0.0008 ***
A–Temperature	3.28	1	3.28	63.09	0.0005 ***
B–Pressure	0.12	1	0.12	2.31	0.1889 n.s.
C–Time	0.17	1	0.17	3.35	0.1267 n.s.
AB	1.25	1	1.25	24.15	0.0044 **
AC	0.20	1	0.20	3.90	0.1053 n.s.
BC	0.0001	1	0.0001	0.0019	0.9667 n.s.
A^2	8.87	1	8.87	170.79	< 0.0001 ***
B^2	0.23	1	0.23	4.44	0.0889 n.s.
C^2	0.037	1	0.037	0.71	0.4376 n.s.
Residual	0.26	5	0.052		
Lack of fit	0.259	3	0.086	215.75	0.0046 **

Cor Total	14.22	14			

Table S3. ANOVA for the porosity (X_v) quadratic regression model of the Box–Behnken hot-pressing optimization. As for Table S2, this is a standard regression ANOVA based on the complete 15-run data set (Table S1); the F values, degrees of freedom, and p values are inferential statistics for the regression model itself. $X_v = 0.77 - 0.64\,A - 0.12\,B - 0.15\,C + 0.56\,AB + 0.23\,AC - 0.01\,BC + 1.55\,A^2 + 0.25\,B^2 + 0.10\,C^2$. $R^2 = 0.9817$, adjusted $R^2 = 0.9489$. Lack-of-fit for X_v is significant ($P = 0.0046$), attributed to the very small pure error of the porosity data; the model diagnostic plots in Figure S3 confirm that the residuals are approximately normal and the predicted vs. actual fit is acceptable for the present process-screening purpose.

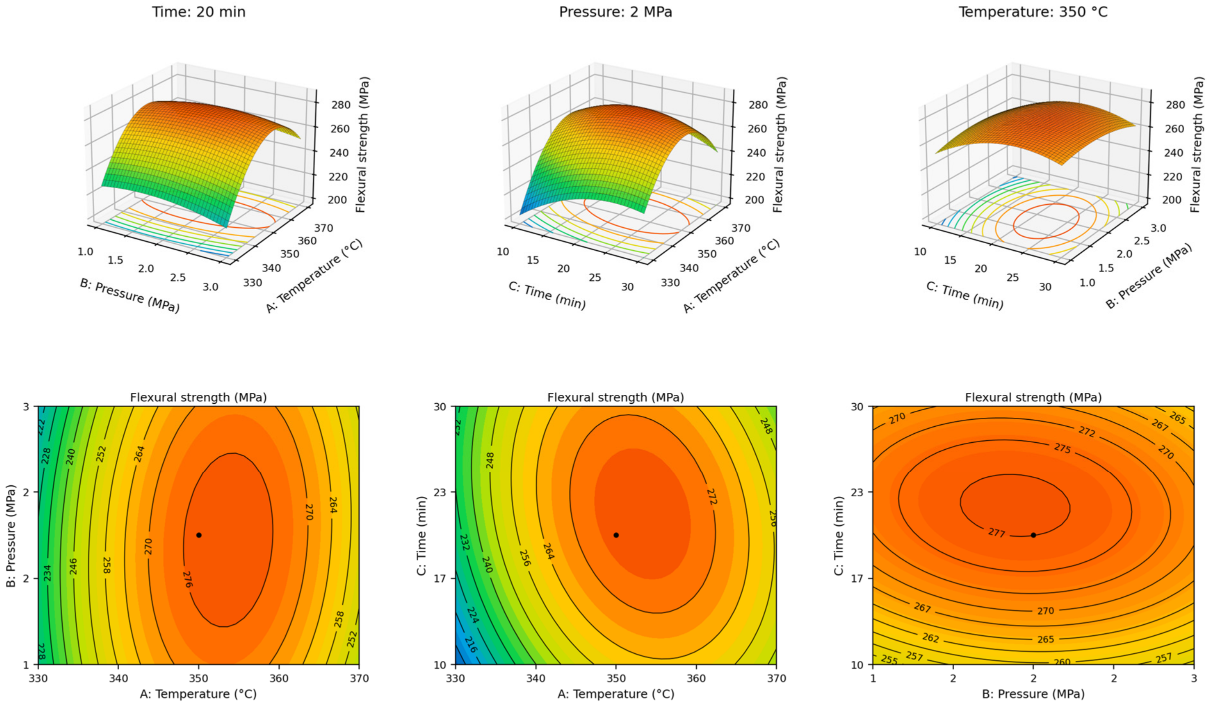

Figure S1. Box–Behnken response surface and contour plots of flexural strength (σ_f). Top row: 3D response surfaces at the center point of the third factor (“Time: 20 min”, “Pressure: 2 MPa”, “Temperature: 350 °C”). Bottom row: corresponding 2D contour plots of the same factor pairs. The recommended window of 345–355 °C / 1.5–2.5 MPa / 15–25 min is recovered as the high-strength plateau in each panel.

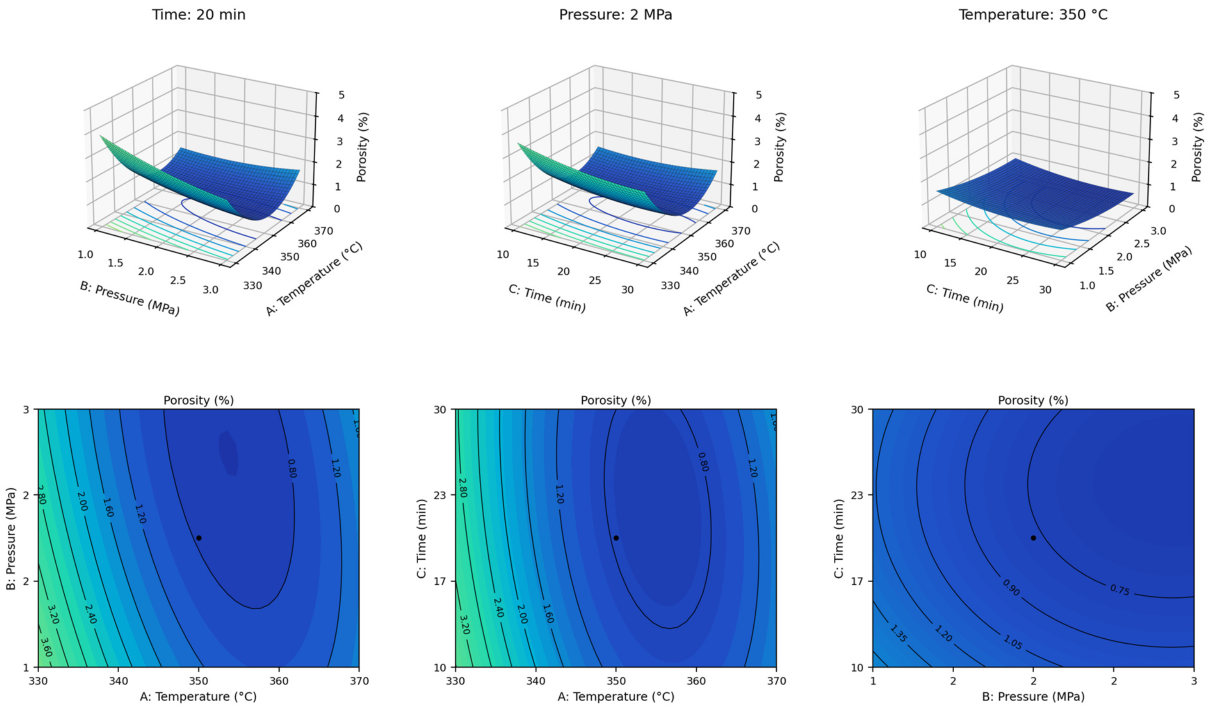

Figure S2. Box–Behnken response surface and contour plots of porosity (X_v). Top row: 3D response surfaces at the center point of the third factor (“Time: 20 min”, “Pressure: 2 MPa”, “Temperature: 350 °C”). Bottom row: corresponding 2D contour plots of the same factor pairs. The minimum-porosity region (≤ 1%) is recovered inside the 345–355 °C / 1.5–2.5 MPa / 15–25 min window, consistent with the recommended flexural-strength window in Figure S1.

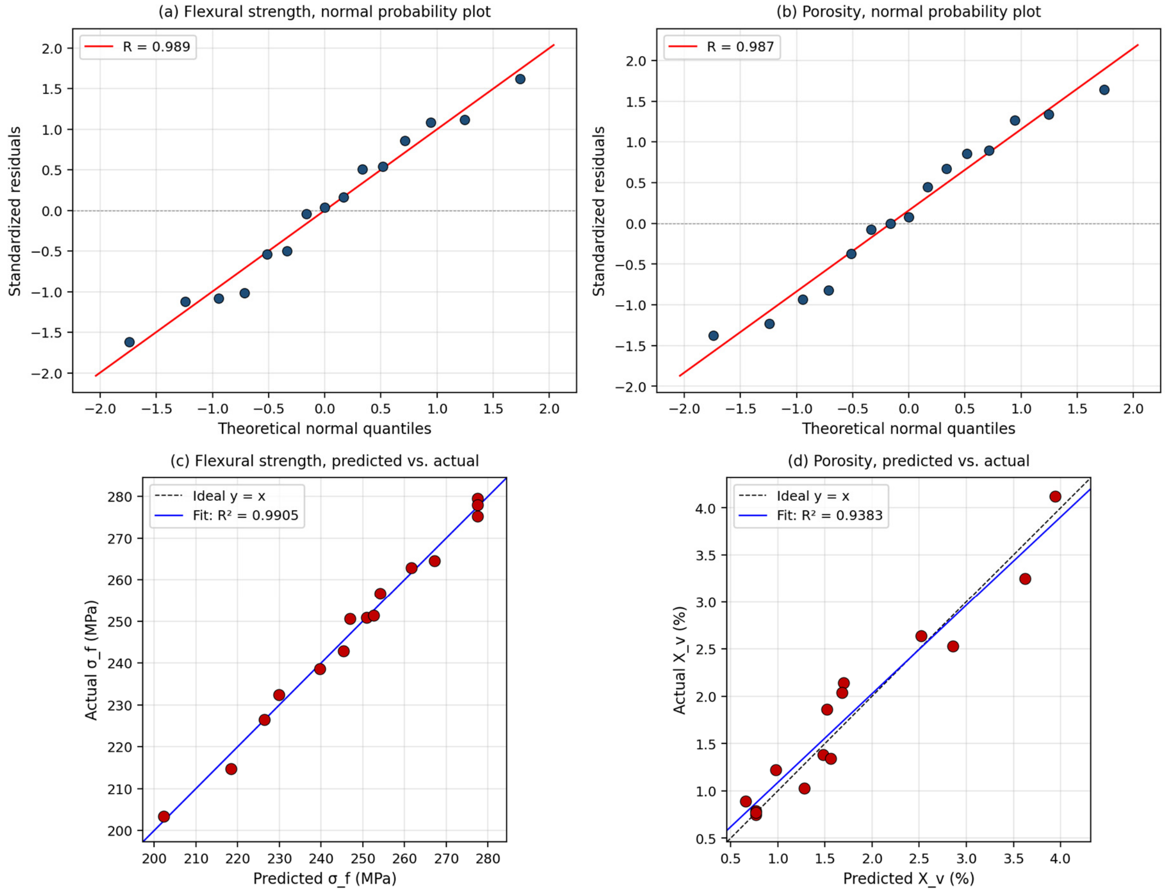

Figure S3. Box–Behnken model diagnostic plots. Top row: normal probability plots of the standardized residuals of the 15-run data. Bottom row: predicted vs. actual values. The flexural strength model ($R^2 = 0.9905$) shows residuals

consistent with a normal distribution (R = 0.989) and an essentially perfect predicted-vs-actual fit. The porosity model (R^2 = 0.9817 on the 15 runs; predicted-vs-actual R^2 = 0.938) shows a small but consistent deviation in the high-porosity region (Run 4: 330 °C / 2 MPa / 10 min, X_v = 3.25%) and a residual distribution that is approximately normal (R = 0.987). These diagnostics support the use of the two quadratic models for the present process-screening purpose despite the statistically significant lack-of-fit of the porosity model (Table S3).

Note: The porosity quadratic model shows significant lack-of-fit (p = 0.0046). This model is provided for descriptive reference only and was not used to select hot-pressing conditions; process window optimization is based solely on the flexural strength response-surface model.

S2. Fiber Content Optimization

Five carbon fiber mass fractions (54.88, 59.34, 64.60, 70.87, 78.49 wt%) were tested under the optimized hot-pressing window (350 °C / 2.0 MPa / 20 min). The theoretical density was calculated from the rule of mixtures as $\rho_{theory} = \rho_{CF} \cdot v_{CF} + \rho_{PEEK} \cdot v_{PEEK}$, where $v_{CF} = (w_{CF}/\rho_{CF}) / (w_{CF}/\rho_{CF} + (1 - w_{CF})/\rho_{PEEK})$ and $v_{PEEK} = 1 - v_{CF}$, with ρ_{CF} = 1.78 g·cm^{-3} and ρ_{PEEK} = 1.30 g·cm^{-3}. The void content was calculated as $X_v = (1 - \rho_{meas}/\rho_{theory}) \times 100\%$. The v_{CF} and X_v values listed in Table S4 follow the unified formula above; the void content shows a U-shaped dependence on the CF content, with a minimum of 0.45% at 54.88 wt% rising to 2.99% at 78.49 wt%.

Both tensile and flexural moduli increased monotonically with fiber content; tensile strength, flexural strength, and ILSS showed a non-linear “increase-then-decrease” pattern with peaks at 59.34–64.60 wt% (Table S5). At 64.60 wt% the porosity was 1.19% and the ILSS reached its maximum of 19.1 MPa. Above 70.87 wt% the porosity rose sharply (1.16% at 70.87 wt% and 2.99% at 78.49 wt%), and the ILSS fell to 17.2 MPa at 70.87 wt% (−9.9% relative to the 64.60 wt% optimum) and to 14.7 MPa at 78.49 wt% (−23.0%). A monotonic correlation between porosity and ILSS was observed across these five compositions (lower porosity associated with higher ILSS); because porosity and fiber content are strongly collinear in this series, this correlation cannot be unambiguously attributed to porosity alone. All specimens in the present interface study were therefore prepared at 350 °C / 2.0 MPa / 20 min with a carbon fiber content of 64.60 wt%.

CF content (wt%)	v_{CF} (%)	ρ_{theory} (g·cm^{-3})	ρ_{meas} (g·cm^{-3})	X_v (%)
78.49	72.72	1.649	1.600	2.99
70.87	63.99	1.607	1.588	1.16
64.60	57.13	1.574	1.555	1.19
59.34	51.60	1.548	1.534	0.89
54.88	47.04	1.526	1.519	0.45

Table S4. Carbon fiber content, fiber volume fraction, theoretical and measured density, and void content for CF/PEEK commingled yarn fabric laminates. Theoretical densities are calculated from the volume fractions of CF and PEEK with the SPEEK sizing mass fraction included in the polymer phase, with ρ_{CF} = 1.78 g·cm^{-3} and ρ_{PEEK} = 1.30 g·cm^{-3}. Measured densities are the mean of three replicate specimens per composition (SD ≤ 0.005 g·cm^{-3}). The v_{CF} column is the carbon-fiber volume fraction calculated from w_{CF} and the theoretical density; X_v is the void content ($X_v = (1 - \rho_{meas}/\rho_{theory}) \times 100\%$) calculated from the same theoretical density. The U-shaped dependence on CF content shows an X_v minimum (0.45%) at 54.88 wt%, rising to 2.99% at 78.49 wt%.

CF content (wt%)	ILSS (MPa)	Tensile strength (MPa)	Tensile modulus (GPa)	Flexural strength (MPa)	Flexural modulus (GPa)
78.49	14.7	245.2	63.60	226.4	61.61
70.87	17.2	264.7	57.32	253.9	56.32
64.60	19.1 (peak)	329.2 (peak)	50.04	277.9 (peak)	57.58
59.34	18.85	300.7	47.91	272.5	50.43
54.88	18.3	293.4	40.21	272.5	40.20

Table S5. Carbon fiber content vs. mechanical properties for CF/PEEK commingled yarn fabric laminates (n = 5).

S3. FTIR Peak Assignments

The FTIR peak assignments used in the main text follow the literature consensus for SPEEK and are summarized in Table S6.

Wavenumber (cm^{-1})	Assignment	PEEK	SPEEK
3060–3100	Aromatic C–H stretch	Present	Present (retained)
1659	C=O carbonyl stretch (PEEK backbone)	Present	Present (retained)
1600, 1496	Aromatic skeletal stretch	Present	Present (retained)
1368	Aromatic C–H bending	Weak	Clear (intensified)
1080	–SO□H S=O stretch	Absent	Present (new)
1020	C–S stretch	Absent	Present (new)
≈ 700	–SO□H S–O symmetric stretch	Absent	Present (new)

Table S6. FTIR peak assignments for PEEK and SPEEK.

S4. Thermal Properties (TGA)

Thermogravimetric analysis (TGA, Q500, TA Instruments) was carried out from 50 to 800 °C at 10 °C·min^{-1} under N_2 on 5–10 mg samples of pristine SPEEK powder, the commercial epoxy sizing, and the composite-sized laminates, to verify that the SPEEK and SPEEK/nanofiller sizings survive the 350 °C / 20 min hot-pressing window. The results are summarized in Table S7.

Code	Sizing	T_5% (°C)	T_max (°C)	Char yield at 600 °C (%)
CF-0	Untreated (epoxy)	507.4	530.1	85.7
CF-B	Acetone-desized	504.4	531.9	84.1
CF-1	SPEEK 0.0025 g·mL^{-1}	n.d.	n.d.	n.d.
CF-2	SPEEK 0.005 g·mL^{-1}	n.d.	n.d.	n.d.
CF-3	SPEEK 0.010 g·mL^{-1}	512.6	533.7	85.5
CF-GO-1	SPEEK/GO 0.5 g·L^{-1}	568.1	573.2	89.0
CF-GO-2	SPEEK/GO 1.0 g·L^{-1}	568.4	575.5	88.5
CF-GO-3	SPEEK/GO 2.0 g·L^{-1}	558.5	571.0	87.7
CF-CNT-1	SPEEK/CNT 0.5 g·L^{-1}	560.7	571.0	89.4
CF-CNT-2	SPEEK/CNT 1.0 g·L^{-1}	561.1	572.0	87.3
CF-CNT-3	SPEEK/CNT 2.0 g·L^{-1}	554.3	566.9	88.7

Table S7. Thermogravimetric characteristics of the eleven CF/PEEK specimens under N_2. T_5% is the 5% mass-loss temperature; T_max is the maximum-rate decomposition temperature; char yield is the residual mass at 600 °C. The

composite-sized specimens (CF-GO and CF-CNT) show a ≈ 50–60 °C increase in T_5% relative to the commercial epoxy baseline (CF-0), confirming that the SPEEK and SPEEK/nanofiller sizings survive the 350 °C / 20 min hot-pressing window. n.d. = not determined (CF-1 and CF-2 were not characterized in the present TGA campaign).

S5. Descriptive Statistics of the GO and CNT Series

The following table summarizes the descriptive statistics (mean ± SD and the relative magnitude of the between-group difference vs. the within-group scatter) for the most contested comparisons in the present data set. The values are reported as a descriptive reference for the present sample set; the n = 5 replicates for each group are test specimens cut from a single consolidated laminate per condition (see Table S8 caption), and the conclusions of the present manuscript rest on the absolute magnitudes of the observed differences and on the consistency of the trends across concentrations rather than on any single p value.

Comparison	Mean A ± SD	Mean B ± SD	Δ (B–A)	Cohen's d (reference)	Direction observed in the present sample set
CF-GO-3 vs CF-CNT-3, ILSS (MPa)	43.2 ± 2.58	48.3 ± 2.39	+5.1	+2.05	CNT > GO (large effect)
CF-GO-3 vs CF-CNT-3, tensile strength (MPa)	586.4 ± 20.5	642.4 ± 23.6	+56.0	+2.53	CNT > GO (large effect)
CF-GO-3 vs CF-CNT-3, tensile modulus (GPa)	81.4 ± 3.05	82.6 ± 1.34	+1.2	+0.51	No clear separation (within scatter)
CF-GO-3 vs CF-CNT-3, flexural strength (MPa)	465.0 ± 31.9	492.3 ± 41.2	+27.3	+0.74	No clear separation (within scatter)
CF-GO-3 vs CF-CNT-3, flexural modulus (GPa)	69.1 ± 4.40	60.3 ± 1.56	−8.8	−2.67	GO > CNT (large effect)
CF-CNT-2 vs CF-CNT-3, tensile modulus (GPa)	87.1 ± 5.69	82.6 ± 1.34	−4.5	−1.09	Plateau; CNT-2 nominally higher (within scatter)
CF-GO-2 vs CF-GO-3, tensile modulus (GPa)	67.1 ± 1.15	81.4 ± 3.05	+14.3	+6.20	GO-3 > GO-2 (very large effect)
CF-CNT-1 vs CF-CNT-2 vs CF-CNT-3, ILSS (MPa)	36.2 → 43.3 → 48.3	—	+12.1	+4.88	Monotonic increase with concentration (range vs. pooled SD)
CF-GO-1 vs CF-GO-2 vs CF-GO-3, ILSS (MPa)	35.2 → 38.2 → 43.2	—	+8.0	+4.65	Monotonic increase with concentration (range vs. pooled SD)

Table S8. Effect-size summary of the most contested comparisons in the GO and CNT series. The sampling design is fundamentally different from the Box–Behnken design in Tables S2–S3: the 15 Box–Behnken runs are 15 independent laminates (one freshly hot-pressed plate per run, so the quadratic regression ANOVA with F and p values in Tables S2 and S3 is a valid inferential test for the regression model), whereas the present table compares 11 formulations, each represented by a single laminate from which 5 test specimens are cut. Cohen's d is computed as (mean_B − mean_A) / pooled within-group SD, where pooled SD = $\sqrt{[(SD\_A^2 + SD\_B^2)/2]}$; values above 0.8 are conventionally regarded as a 'large' effect and above 2.0 as a 'very large' effect (Cohen, 1988). The n = 5 replicates for each group are test specimens cut from a single representative consolidated laminate per condition, so the within-group SD reflects specimen-to-specimen variation within one plate rather than plate-to-plate variation; the d values are therefore descriptive of the present sample set only and are not used as inferential statistics on the CF/PEEK population, because the within-plate sampling design does not satisfy the independence assumption of inferential ANOVA. A confirmatory study with n = 3–5 independent plates per group and a nested analysis with plate as a random factor is the subject of ongoing work.

Sample	Sizing	M_f(wt%)	V_f(vol%)	ρ_{theory} ($g\cdot cm^{-3}$)	$\rho_{measured}$ ($g\cdot cm^{-3}$)	porosity (%)
CF-0	Untreated (epoxy)	59.72±0.01	51.99±0.01	1.549	1.537±0.002	0.76
CF-B	Acetone-desized	-				-
CF-3	SPEEK 0.010 g/mL	59.86±0.02	52.13±0.02	1.550	1.535±0.003	0.97
CF-GO-1	SPEEK/GO 0.5 g/L	-				-
CF-GO-2	SPEEK/GO 1.0 g/L	59.69±0.01	51.96±0.01	1.549	1.531±0.004	1.16
CF-GO-3	SPEEK/GO 2.0 g/L	60.76±0.03	53.07±0.03	1.555	1.540±0.003	0.94
CF-CNT-1	SPEEK/CNT 0.5 g/L	60.44±0.03	52.74±0.03	1.553	1.538±0.003	0.96
CF-CNT-2	SPEEK/CNT 1.0 g/L	60.77±0.01	53.08±0.01	1.555	1.539±0.002	1.00
CF-CNT-3	SPEEK/CNT 2.0 g/L	-			-	-

Table S9. The fiber content and porosity of the CF/PEEK samples were determined by acid digestion method. Porosity measurements are not available for CF-B, CF-GO-1 and CF-CNT-3 in the current dataset. Mf = carbon-fiber mass fraction; Vf = carbon-fiber volume fraction; ρtheory = theoretical laminate density; ρmeasured = experimentally measured density.

Note s1

Sulfonation degree by acid–base titration. The DS of the SPEEK overlayer was determined by dissolving ≈ 0.10 g of SPEEK in 20 mL of DMF, adding 2–3 drops of phenolphthalein indicator, and titrating against a standardized 0.01 mol·L^{-1} NaOH in DMF solution until the color change persisted for 30 s. Three parallel titrations gave DS = 0.78 ± 0.02. The DS is calculated from the equivalent of NaOH consumed per gram of SPEEK using the relation DS = (M_SPEEK × n_NaOH) / (m_SPEEK − 80 × n_NaOH), where M_SPEEK is the repeat-unit molecular weight and 80 g·mol^{-1} is the net mass change per sulfonic-acid group; the SPEEK blank was run under the same conditions and the consumption was negligible.
